# Precise Regulation of Membrane Proteins: From Physical Technology to Biomolecular Strategy

**DOI:** 10.1002/advs.202510367

**Published:** 2025-12-19

**Authors:** Xiu Zhao, Qingqing Zhou, Mengli Li, Wen Zou, Zhenzhong Zhang, Jinjin Shi, Junjie Liu

**Affiliations:** ^1^ School of Pharmaceutical Sciences Zhengzhou University Zhengzhou Henan 450001 China; ^2^ Henan Key Laboratory of Nanomedicine for Targeting Diagnosis and Treatment Zhengzhou University Zhengzhou Henan 450001 China; ^3^ Pingyuan Laboratory State Key Laboratory of Antiviral Drugs

**Keywords:** biomolecules, disease treatments, membrane protein regulations, physical technologies

## Abstract

As essential functional components of the cell membrane, membrane proteins serve as one of the key mechanisms for sensing external stimuli and play crucial roles in diverse biological processes, including cell migration, proliferation, differentiation, and apoptosis. These processes are closely related to the maintenance of physiological homeostasis and the progression of various diseases. In recent years, with an in‐depth understanding of membrane protein functions, developing membrane protein regulation strategies has emerged as a significant direction in both basic research and therapeutic applications. Precise modulation of membrane protein expression levels, conformational changes, and spatial distribution enables artificial regulation of cellular behavior, thereby opening promising avenues for treating diseases such as cancer, neurodegenerative disorders, and immune dysfunctions. This review comprehensively summarizes various physical technologies and biomolecule‐driven approaches for membrane protein regulation, including applying light/temperature/magnetism/ultrasound‐sensitive modules to achieve external physical control, as well as biomolecular tools, such as DNA, peptides, and proteins with specific receptor recognition properties that modulate membrane proteins via intermolecular interactions. Furthermore, the advantages and limitations of different membrane protein regulation techniques are discussed, aiming to provide new insights into cell function regulation and disease treatment.

## Graphical abstract

This review summarizes the emerging strategies for the precise regulation of membrane proteins using physical stimuli and biomolecule‐based tools. These methods provide new insights into cell regulation and offer promising directions for future disease treatment.

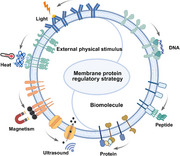

## 1 Introduction

Membrane proteins, which span or are embedded in biological membranes, play critical roles in numerous essential biological processes. These include maintaining cellular structural integrity, mediating substance exchange, facilitating signal transduction, and enabling intercellular communication.^[^ ^1^, ^2^ ^]^ Studies have demonstrated that membrane proteins enable the transport of nutrients, metabolites, and ions across the phospholipid bilayer. Furthermore, receptor membrane proteins, such as the tyrosine kinase family, orchestrate signal transduction processes that regulate growth, differentiation, and metabolism. In addition, certain membrane proteins participate in intercellular interactions and mediate cell‐cell recognition, as exemplified by immune cells’ specific recognition of antigens.^[^ ^3^, ^4^ ^]^ It is reported that membrane proteins encoded by the human genome account for ≈25% of the total proteome, and ≈50% of these are recognized drug targets.^[^ ^5^, ^6^ ^]^ Based on the membrane‐binding modes and functional characteristics, membrane proteins are commonly classified into three categories: 1) Peripheral membrane proteins, including enzymes, receptor proteins, and intercellular junction proteins, which are primarily involved in cell signal transduction, immune responses, and intercellular interactions; 2) Transmembrane proteins (≈70–80% of membrane proteins), including transmembrane ion channel proteins, G protein‐coupled receptors, and receptor tyrosine kinases (RTKs), responsible for material transport and signal amplification; 3) Lipid‐anchored membrane proteins, attached to membranes via lipid moieties, which contribute to signal transduction and the regulation of membrane stability.^[^ ^7^ ^]^

**Figure 1:**
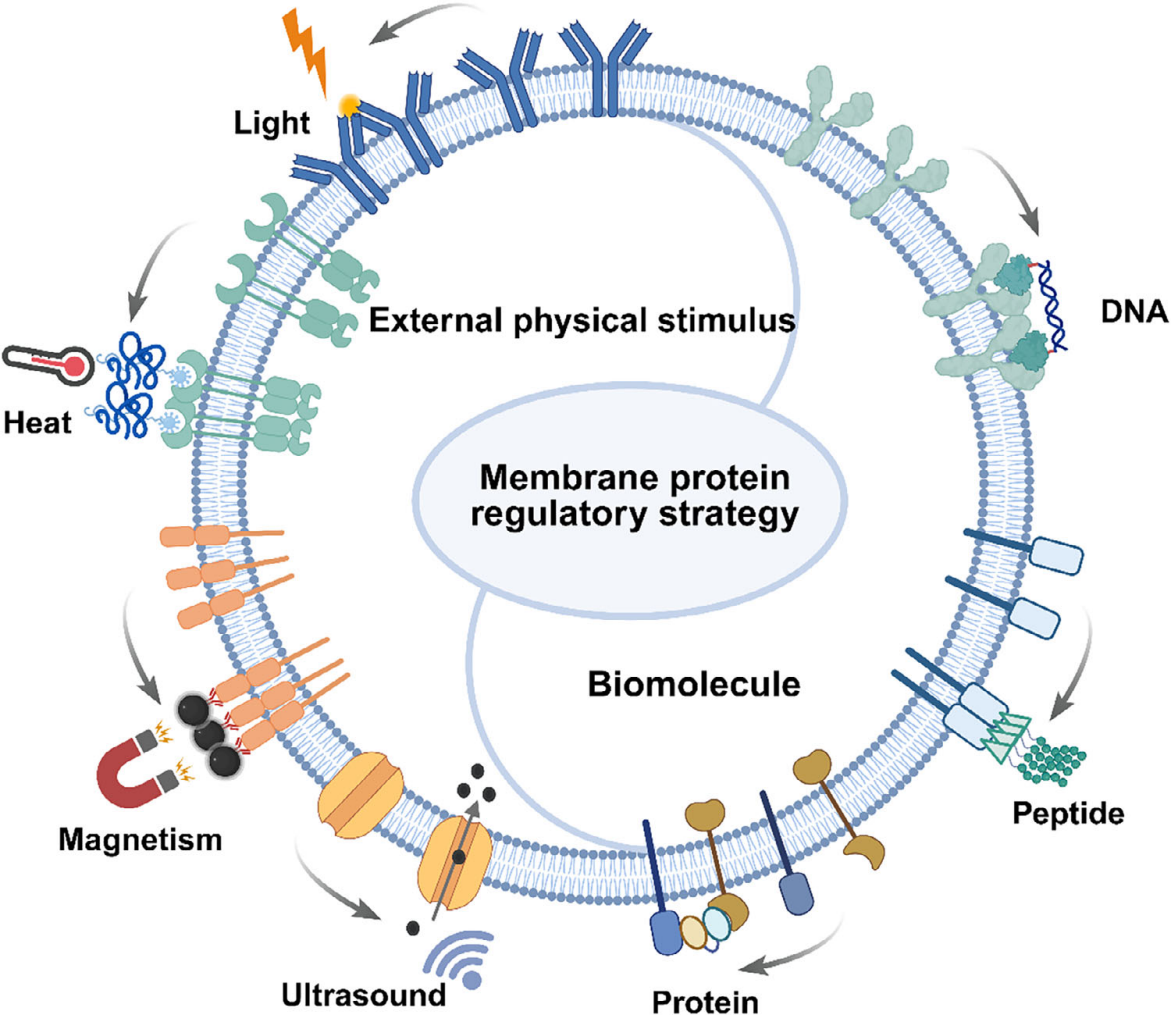
Schema of physical technology and biomolecule‐mediated membrane protein regulation strategies. The figure was created using BioRender (biorender.com).

In view of the vital impact of membrane proteins on cellular functions, their dysfunction is frequently associated with various diseases, including diabetes, cardiovascular disease, and neurodegenerative diseases.^[^ ^8^ ^]^ Consequently, researchers have increasingly focused on developing strategies to modulate membrane proteins for providing novel therapeutic approaches.^[^ ^9^, ^10^, ^11^ ^]^ In this review, we mainly summarize the recent advances in membrane protein regulation strategies that are independent of gene editing technology. These approaches are broadly divided into two categories according to the functional components employed: 1) physical technology‐mediated strategies: which utilize external stimulus‐responsive units to achieve precise regulation of membrane proteins by modulating the parameters of external physical fields, such as light, temperature, magnetism, and ultrasound; 2) biomolecule‐mediated strategies: which employ biomolecules including DNA, peptides, and proteins as functional groups to regulate membrane proteins through programmable assembly and molecular recognition (**Figure** **1**). This work aims to provide new insights into the precise regulation of membrane proteins, offering a theoretical foundation and practical guidance for developing novel therapeutic strategies.

## 2 Physical Technology‐Mediated Membrane Protein Regulation Strategies

### 2.1 Light Regulation

Light serves as an ultrafast trigger with response times ranging from microseconds to milliseconds, enabling highly precise signal output.^[^ ^12^ ^]^ These characteristics make it particularly suitable for the non‐invasive and controllable regulation of membrane proteins, thereby offering crucial technical support for developing related disease treatments.^[^ ^13^, ^14^, ^15^, ^16^, ^17^, ^18^ ^]^ It is noteworthy that optogenetics has also been widely applied to membrane protein regulation, which was systematically reviewed elsewhere.^[^ ^19^, ^20^ ^]^ This section mainly focuses on physical regulation strategies based on light‐responsive molecules or materials. By introducing photo‐responsive functional groups, these materials can undergo isomerization, photolysis, or photothermal effect under different wavelengths of irradiation, thereby achieving high spatiotemporal control over anchored membrane proteins.

**Figure 2:**
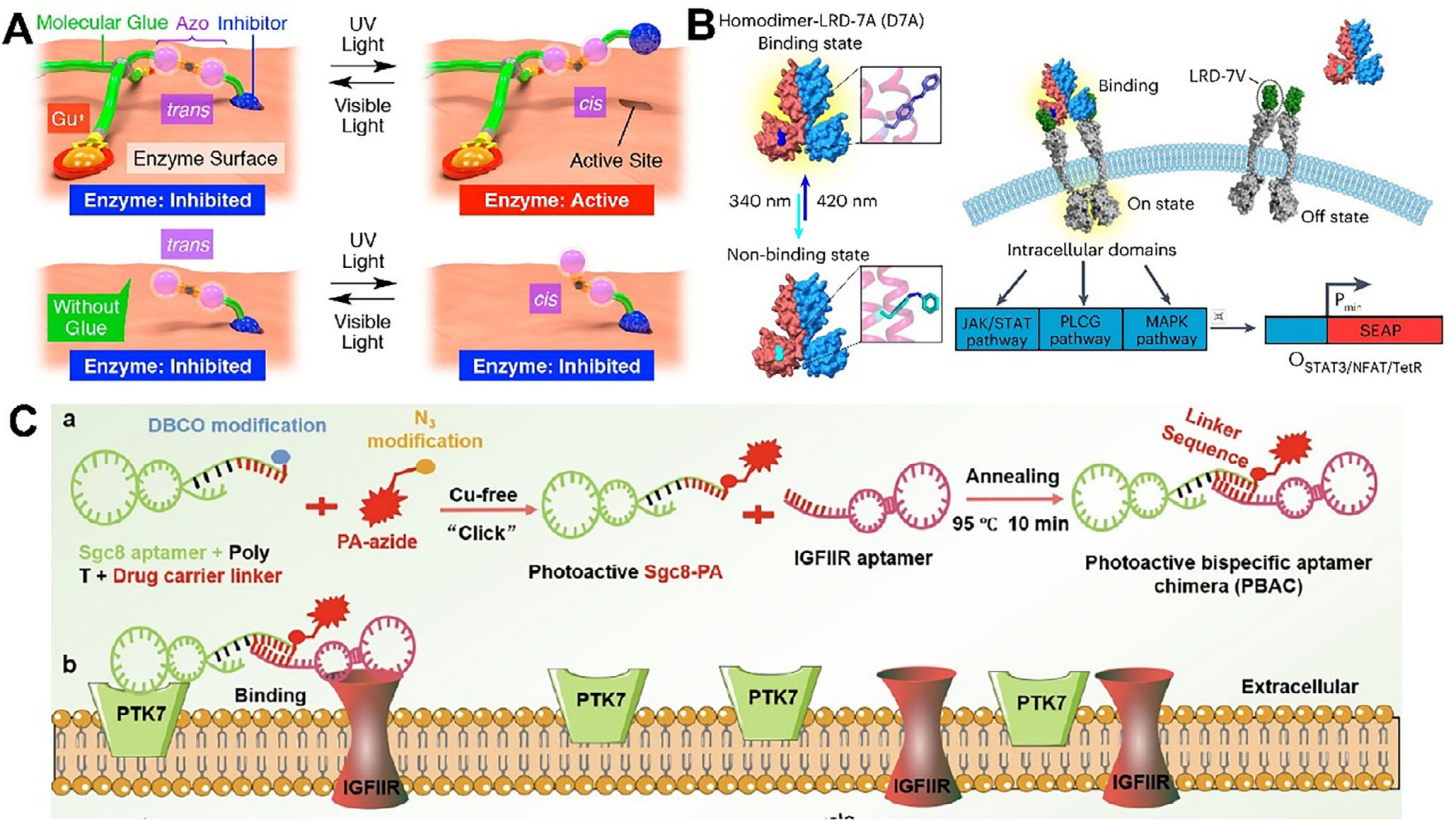
Light‐mediated regulation of membrane proteins. A) An Azo‐based photoswitch mediated photochemical regulation of CA enzymes. Reproduced with permission.^[^ ^27^ ^]^Copyright 2017, Journal of the American Chemical Society. B) A photoresponsive ligand D7A based on AzoF enabled reversible dimerization of receptors. Reproduced with permission.^[^ ^29^ ^]^ Copyright 2025, Nature Chemistry. C) NIR light‐activated lysosome‐targeting chimera constructed from a photoactive bispecific aptamer conjugate for precise degradation of the membrane protein PTK7. Reproduced with permission.^[^ ^31^ ^]^ Copyright 2025, Journal of the American Chemical Society.

Among various photo‐responsive motifs, azobenzene (Azo) derivatives are the most widely employed.^[^ ^21^, ^22^, ^23^, ^24^, ^25^ ^]^ Upon illumination, Azo undergoes cis‐trans isomerization, enabling light‐dependent tuning of ligand affinity toward the receptor. By conjugating Azo with protein inhibitors or activators, reversible modulation of protein function can be achieved. For example, the Trauner team synthesized a novel Azo propofol derivative (AP2), which could reversibly undergo photo‐controlled transformation between active and inactive conformations.^[^ ^26^ ^]^ AP2 had a high affinity for Gamma‐aminobutyric acid type A receptor (GABA_A_) receptors *under* dark conditions and could significantly enhance GABA_A_‐induced Cl^−^ currents. Under illumination at 390–450 nm, the enhancement effect was suppressed considerably, indicating that AP2 could serve as a potent tool molecule for light‐controlled GABA_A_ receptor signaling. Beyond receptor proteins, enzymes are critical for maintaining intracellular stability and function, making them attractive targets for photoregulation. Aida et al. developed a water‐soluble adhesive photoswitch composed of an Azo unit, an enzyme‐inhibiting sulfonamide (SA) motif, and a guanidinium ions (Gu^+^) functionalized gel segment (Gluen) (**Figure** **2**A).^[^ ^27^ ^]^ The SA moiety specifically bound to carbonic anhydrase (CA) and inhibited its activity, while guiding the linked Gluen part to an oxyanion‐rich region near the active site, thereby ensuring stable conjugation between photoswitch and CA. The photochemical isomerization of the Azo unit induced a push‐pull movement of SA, resulting in its undocking from the CA active site. Similarly, Liu and coworkers fabricated enzyme nanocapsules by combining an Azo‐modified monomer (Acr‐Azo‐SA) with CA.^[^ ^28^ ^]^ The acrylate (Acr) group enabled Azo‐SA to participate in free radical polymerization, allowing it to be firmly anchored within the polymer network and stably positioned near the enzyme active site. This design enabled reversible, photo‐controlled regulation of enzymatic activity. This strategy was applicable not only to CA but also to acetylcholinesterase (AChE). In addition, Cao and coworkers employed the noncanonical amino acid azophenylalanine (AzoF) to design a reversible light‐controlled protein‐protein interaction system (Figure 2B).^[^ ^29^ ^]^ In this system, the AzoF‐containing chain LRD‐7A was coupled to a homodimer via a flexible linker to form the ligand D7A, which specifically bound to the receptor chain LRD‐7 V. Under 420 nm or ambient light, AzoF remained in the trans‐conformation, allowing D7A to induce receptor dimerization and activate downstream signaling. Under 340 nm irradiation, AzoF switched to the cis conformation, causing ligand‐receptor dissociation and signal shutdown.

Compared with isomerization‐based photoswitches, photocleavable molecules enabled more direct regulation through bond cleavage. For instance, Aida et al. developed a dendritic molecular glue, ^PC^Glue‐NBD, by incorporating a photocleavable bond.^[^ ^22^ ^]^ This construct firmly adhered to the target protein via multivalent salt bridge interactions between its Gu⁺ groups and the oxygen‐containing anion group of hepatocyte growth factor (HGF). Simultaneously, its large dendritic wedge inhibited interactions between HGF and the cellular‐mesenchymal‐epithelial transition factor (c‐Met). Upon ultraviolet light (UV) irradiation, ^PC^Glue‐NBD underwent cleavage at the carbamate bond, releasing HGF and restoring its intrinsic affinity for c‐Met. This led to c‐Met dimerization and subsequent cell migration. In addition, the Tampe team engineered a high‐affinity lock‐and‐key pair (trisNTA/His) based on biotinylated photoactivatable trivalent nitrilotriacetic acid for regulating the distribution of neuropeptide Y_2_ receptor (Y_2_R) on HeLa cells.^[^ ^30^ ^]^ This design comprised two key elements: 1) a trivalent azatriacetic acid (trisNTA) chelator immobilized on a 2D matrix for specific binding to His_6_‐tag, and 2) a His_6_‐tag genetically fused to the N‐terminus of the Y_2_R. The trisNTA activity was auto‐inhibited through interaction with the His_6_‐tag. Upon 405 nm laser irradiation, the chemical structure of trisNTA was altered, causing it to lose binding ability for the His tag, thereby releasing Y_2_R to aggregate on the membrane and promoting cell migration.

Apart from UV‐responsive materials, near‐infrared (NIR) light‐mediated photosensitive groups have been widely utilized for the membrane proteins degradation and therapeutic interventions. Zhang et al. constructed a NIR light‐activated lysosome‐targeting chimera based on a photoactive bispecific aptamer conjugate (PBAC), enabling precise degradation of the membrane protein tyrosine kinase 7 (PTK7) (Figure 2C).^[^ ^31^ ^]^ The PBAC was assembled from three components: the Sgc8 aptamer targeting PTK7, an aptamer recognizing the lysosomal receptor insulin‐like growth factor II receptor (IGFIIR), and a NIR‐sensitive photoactive module, pheophorbide A (PA). Through dual‐specific recognition, this system directed PTK7 into the lysosome. Upon NIR irradiation, the PA module induced oxidative stress and autophagy, synergistically promoting degradation of the membrane protein. In addition, NIR photoimmunotherapy (NIR‐PIT) has emerged as a novel light‐controlled strategy for membrane protein modulation.^[^ ^32^ ^]^ By covalently conjugating a photosensitizer such as IR700 to a monoclonal antibody that targets specific membrane proteins, this approach enabled selective destruction of cancer cells. Under 690 nm NIR irradiation, IR700 underwent a photochemical reaction that triggered the aggregation of antibody‐membrane protein complexes and caused localized membrane damage, leading to necrosis‐like cell death. This strategy had been applied to various membrane proteins, including epidermal growth factor receptor (EGFR), human epidermal growth factor receptor 2 (HER2), CD44, and CD133.^[^ ^33^, ^34^, ^35^, ^36^ ^]^

In addition, photothermal‐responsive materials capable of converting light energy into heat are also commonly employed to regulate membrane proteins. For example, Cheng and coworkers synthesized ultrasmall platinum nanoparticles (PtNPs) inside various enzyme molecules, including glucoamylase (GA), using a simple chemical method.^[^ ^37^ ^]^ Leveraging the efficient photothermal conversion property of PtNPs, pulsed NIR light could locally raise the temperature of the enzyme molecules, thus enhancing the catalytic efficiency of enzyme active center. In another study, Yoo et al. designed NIR‐sensitive gold nanorods (GNR) as nanoscale photothermal transducers.^[^ ^38^ ^]^ Modification with amine‐terminated polyethylene glycol (NH_2_‐PEG) enabled GNRs to target negatively charged neuronal membranes via electrostatic interaction. Under 785 nm irradiation, GNRs converted light energy into local heat, activating the thermosensitive potassium channel TREK‐1 and suppressing neuronal firing, thus serving as effective tools for brain disease therapy. Similarly, Wen et al. conjugated copper sulfide (CuS) nanoparticles with TRPV1 monoclonal antibodies to construct a photothermal switch (CuS‐TRPV1) targeting vascular smooth muscle cells (VSMCs).^[^ ^39^ ^]^ Under 980 nm NIR light, local heating activated TRPV1 channels on the VSMC membrane, leading to Ca^2^⁺ influx, inhibiting foam cell formation, and intervening in atherosclerosis progression. Furthermore, the Salaita group developed an optomechanical actuator (OMA) that modulated the receptor under low‐intensity NIR illumination.^[^ ^40^ ^]^ The OMA nanoparticles comprised gold (Au) nanorods coated with a thermoresponsive polymer shell (poly(N‐isopropylmethacrylamide), which could be functionalized with protein ligands.^[^ ^62^ ^]^ The Au nanorods served as photothermal transducers, converting NIR pulses into localized heat to induce rapid contraction of the polymer shell. This contraction exerted mechanical force on the tethered ligands, initiating actin polymerization and ultimately promoting cell migration.

### 2.2 Temperature Regulation

Temperature‐based regulation, as an extension of photothermal effects, offers a partial solution to the limited tissue penetration of light. However, this approach generally exhibits lower response speed and spatial resolution compared to optical methods. In recent years, numerous studies have focused on developing temperature‐sensitive polymers with good biocompatibility.^[^ ^41^, ^42^ ^]^

**Figure 3:**
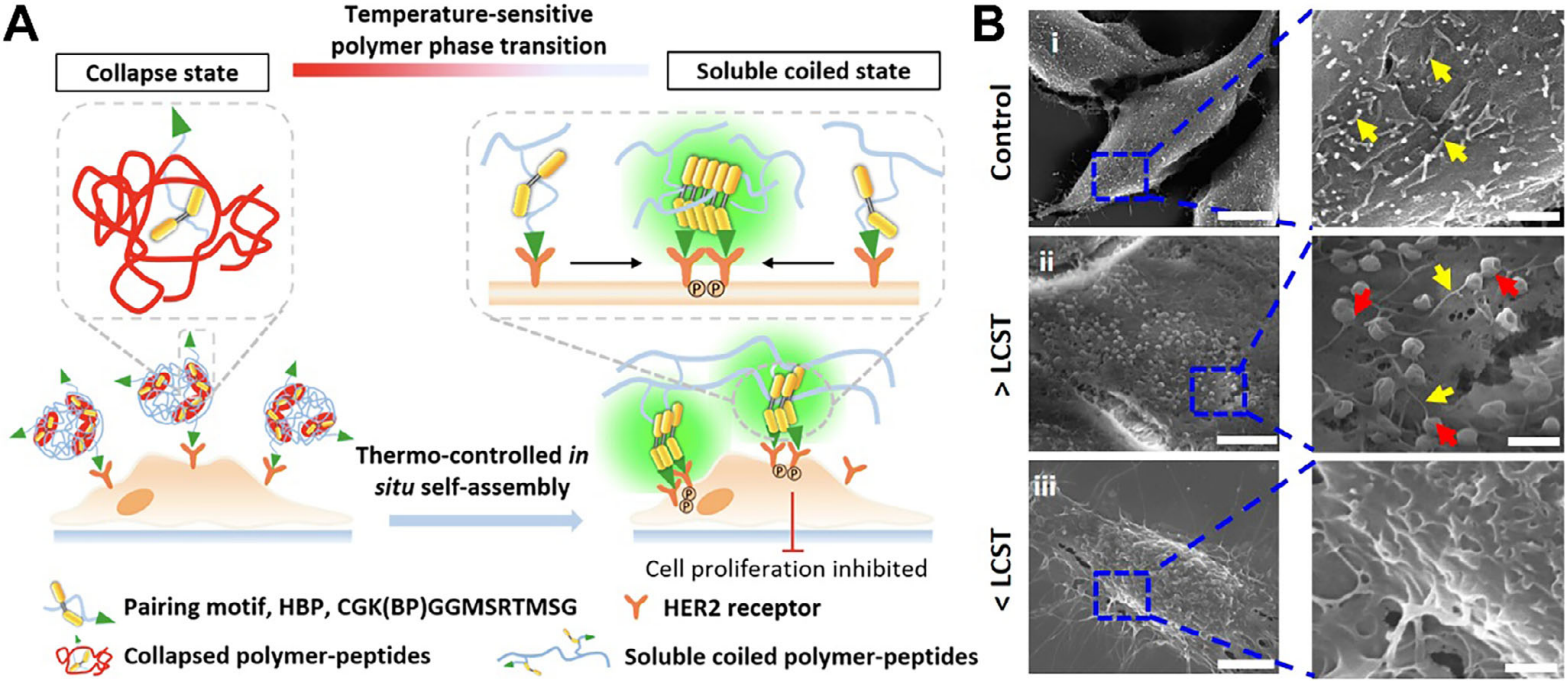
Temperature‐mediated membrane proteins regulation. A) A temperature‐controlled strategy manipulated HER2 receptor aggregation through conformational transitions of temperature‐sensitive PNIPAAm polymer. B) SEM images to visualize PBP1 (pyrene‐based polymer 1) structural transition on SK‐BR‐3 (human breast cancer cell line) cell surfaces. (LCST: Lower Critical Solution Temperature) Reproduced with permission.^[^ ^50^ ^]^ Copyright 2016, ACS Applied Materials & Interfaces.

Among these, the temperature‐responsive polymer poly (N‐isopropylacrylamide) (PNIPAAm) has been the most extensively utilized.^[^ ^43^, ^44^, ^45^, ^46^, ^47^, ^48^ ^]^ For example, Hoffman et al. reported an N49C mutant streptavidin featuring a polymer conjugation site introduced near the biotin binding pocket.^[^ ^49^ ^]^ The researchers functionalized PNIPAAm with vinyl sulfone groups via reaction with divinyl sulfone and triethylamine, followed by conjugation to N49C streptavidin. Below 32 °C, the streptavidin retained its ability to bind biotin, whereas above 37 °C, the collapse of the polymer chain inhibited binding. This reversible switching enabled regulation of biotin‐streptavidin interactions through temperature‐controlled conformational changes in the PNIPAAm‐streptavidin conjugate. In another study, the Wang team developed a temperature‐control strategy to manipulate and monitor HER2 receptor aggregation through in situ conformational transitions of polymer conjugates on the cell surface (**Figure** **3**).^[^ ^50^ ^]^ First, a HER2‐targeting peptide was linked to a hydrophobic bispyrene molecule (BP) with aggregation‐induced emission properties, forming a functional motif termed HBP. HBP was then conjugated to PNIPAAm polymer, endowing the HER2‐targeting peptide with temperature‐dependent characteristics. At 40 °C, the PNIPAAm polymer shrank to form a “shield” that prevented HBP aggregation. When the temperature was lowered to 35 °C, the polymer extended and exposed HBP to allow its aggregation, which enhanced fluorescence and promoted HER2 receptor clustering, ultimately leading to cytoplasmic phosphorylation and inhibition of cancer cell proliferation.

### 2.3 Magnetic Regulation

In addition to light and temperature, magnetism represents another important physical cue for regulating membrane proteins. Compared with other remote cell‐manipulation techniques, magnetic technology offers unique advantages. Magnetic fields can penetrate biological tissues with negligible attenuation, making them particularly suitable for in vivo applications.^[^ ^51^, ^52^ ^]^ By functionalizing membrane proteins with magnetic nanoparticles, external magnetic fields can be utilized to regulate membrane proteins and manipulate cell functions.^[^ ^53^, ^54^ ^]^

**Figure 4:**
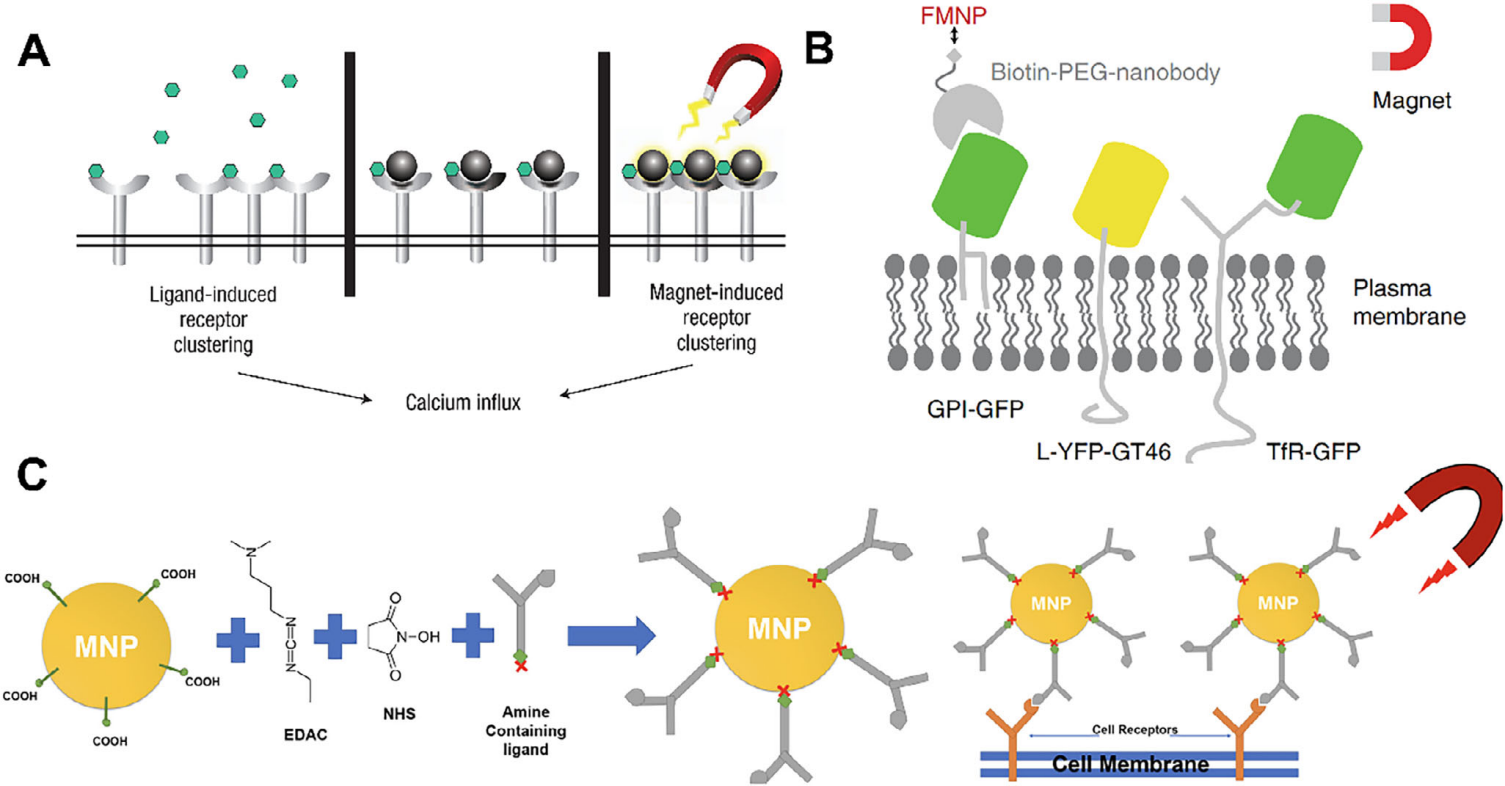
Regulation of membrane proteins via magnetic fields. A) Superparamagnetic nanobeads conjugated with dinitrophenyl‐human serum albumin induced aggregation of FcεRI receptors under the external magnetic field. Reproduced with permission.^[^ ^60^ ^]^ Copyright 2008, Nature Nanotechnology. B) Functionalized magnetic nanoparticles (FMNP) coated with streptavidin enabled manipulate membrane proteins. Reproduced with permission.^[^ ^62^ ^]^ Copyright 2020, Nature Communications. C) Magnetic nanoparticles of antibodies targeting integrin α5 generated mechanical forces under external magnetic field, mimicking extracellular mechanical signals. Reproduced with permission.^[^ ^67^ ^]^ Copyright 2024, Pharmaceutics.

For instance, Schneck et al. conjugated MHC‐Ig dimers and anti‐CD28 antibodies onto the surface of microbeads to construct Nano‐aAPC nanoparticles capable of binding T cell receptors (TCRs).^[^ ^55^ ^]^ Under an external magnetic field, the Nano‐aAPC nanoparticles bound to TCRs were driven to aggregate, promoting TCR clustering and enhancing T cell activation. In a melanoma adoptive immunotherapy model, magnetic field‐assisted activation of T cells via Nano‐aAPC effectively induced tumor regression. Considering that superparamagnetic iron oxide nanoparticles (SPIONs) exhibit negligible magnetic hysteresis.^[^ ^56^, ^57^, ^58^, ^59^ ^]^ Ingber and coworkers fabricated superparamagnetic nanobeads functionalized with multiple dinitrophenyl‐human serum albumin (DNP‐HSA) molecules on their surfaces, which were subsequently bound to FcεRI receptors on RBL‐2H3 mast cells (**Figure** **4**A).^[^ ^60^ ^]^ Exposure to an external magnetic field induced physical aggregation of the nanobead‐receptor complexes on the cell membrane, thus activating downstream signaling pathways. In addition, Jovin et al. conjugated streptavidin onto SPIONs, which were further coupled with biotinylated EGFR monoclonal antibodies to form a “magnetic switch”.^[^ ^61^ ^]^ Under an external magnetic field, SPIONs aggregation triggered EGFR autophosphorylation and downstream signaling, mimicking the effects of ligand‐dependent EGFR activation. Additionally, Li et al. designed fluorescent magnetic nanoparticles (FMNPs) consisting of a superparamagnetic nanoparticle core coated with a fluorescent polymer shell and modified with streptavidin, enabling specific binding to membrane proteins via biotinylated anti‐green fluorescent protein nanobodies (Figure 4B).^[^ ^62^ ^]^ By applying finely controlled femto‐newton (fN)‐scale magnetic forces using a micromagnetic needle under a microscope, FMNP‐labeled membrane proteins, such as glycosylphosphatidylinositol‐anchored proteins, single‐pass transmembrane proteins, and transferrin receptors were precisely manipulated and repositioned on the cell membrane, achieving reversible spatial regulation at the single‐molecule level. However, the applied force remains relatively small (in the fN range), and the precision of magnetic needle positioning limits the technique to adherent cells or model membrane systems, requiring further optimization for deep‐tissue or in vivo applications. To achieve reversible mechanical regulation of membrane proteins, Bian et al. developed a heterodimers‐based switchable system.^[^ ^63^ ^]^ The system consisted of superparamagnetic Fe_3_O_4_ nanocages (MNCs) and gold nanoparticles (AuNPs) functionalized with the arginine‐glycine‐aspartic acid (RGD) peptide. The two components were connected by a flexible thiol‐PEG linker and immobilized on a substrate. Under an external magnetic field, the MNCs were driven to expose the RGD motifs on the AuNP surface to specifically bind integrins on the cell membrane, thereby promoting stem cell adhesion and spreading. Upon removal of the magnetic field, the MNCs returned to their original positions through the elastic recovery of the linker, re‐covering the RGD sites and blocking integrin‐RGD interactions. This process enabled dynamic control over cell adhesion behavior.

Although nanobeads exhibit uniform size and robust surface functionalization capacity, their high cost pose practical constraints. In contrast, magnetic nanoparticles are more cost‐effective and can be synthesized through diverse methods, making them widely applicable.^[^ ^64^, ^65^ ^]^ For example, Marie et al. developed a “stealth” magnetic nanoparticle (syMagIcS), which was composed of a maghemite (MCP) core coated with a self‐assembled monolayer of the fusion protein mEGFP::Mms6ΔN.^[^ ^66^ ^]^ The Mms6ΔN fragment served as an efficient iron‐binding and mineralization template, while the mEGFP β‐barrel structure provided a biocompatible shell with fluorescent labeling capability. Using the high‐affinity interaction between the mEGFP‐αGFP nanobody pair, the syMagIcS nanoparticles were precisely coupled to target proteins tagged with αGFP, the T‐cell lymphoma invasion and metastasis protein 1 (TIAM1). The magnetic field gradient generated by a miniature external magnet drove rapid migration and accumulation of the complex at the plasma membrane, leading to localized enrichment of TIAM1 and activation of the signaling pathway, ultimately regulating actin cytoskeleton rearrangement and cell migration. In addition, Hajiali et al. covalently attached antibodies or specific aptamers targeting integrin α5 onto magnetic nanoparticles surface (Figure 4C).^[^ ^67^ ^]^ Under an external magnetic field, these nanoparticles exhibited directional oscillation or displacement in response to the periodic variation of the field, exerting cyclic microscale mechanical forces on integrin α5 receptors. This mimics the mechanical cues from the extracellular matrix, which are transduced intracellularly via integrin‐mediated mechanotransduction pathways, ultimately promoting osteogenic differentiation of mesenchymal stem cells. The Kang team synthesized Fe_3_O_4_ aggregates of different sizes and immobilized them using flexible linkers onto bioactive materials pre‐functionalized with the cell‐adhesive RGD By remotely controlling the position of the aggregates via an external magnetic field, ligand accessibility was dynamically modulated by altering the distance between the aggregates and the ligands.^[^ ^68^ ^]^ This strategy effectively enhanced macrophage adhesion‐mediated pro‐healing polarization both in vitro and in vivo.

The enhanced magnetism properties of zinc‐doped iron oxide nanoparticles have further expanded their applicability. For instance, the Jun team developed a “mechanogenetics toolkit” technique, which were fabricated by coating zinc‐doped iron oxide magnetic cores with an amino‐functionalized silica layer, followed by deposition of a gold shell.^[^ ^69^ ^]^ By specific ligand‐receptor interactions targeting Notch receptors and VE‐cadherin, and the application of magnetic force, the mechanical signals generated by extracellular matrix or intercellular interactions were mimicked. In another study, Cheon et al. construct an antibody‐magnetic nanoparticle complex (Ab‐MNPs) using zinc‐doped iron oxide magnetic nanoparticles (Zn_0.4_Fe_2.6_O_4_ MNP, 15 nm) functionalized with DR4 antibodies.^[^ ^70^ ^]^ Under an external magnetic field, the Ab‐MNPs accumulated on the cell membrane and induced DR4 receptor clustering, ultimately promoting apoptosi via Fas‐associated death domain and procaspase‐8 recruitment.

### 2.4 Ultrasound Regulation

Ultrasound, as a remote and noninvasive physical stimulus, has emerged as an important complement to light, temperature, and magnetic‐field‐based approaches due to its excellent tissue penetration, spatial controllability, and safety. Its mechanisms of action primarily involve mechanical vibration and microbubble cavitation, which can directly or indirectly alter the membrane tension state as well as the conformation and activity of membrane proteins, thereby enabling reversible modulation of membrane protein function.^[^ ^71^, ^72^, ^73^ ^]^

Sorum et al. first reported that ultrasound could activate the mechanosensitive potassium channel TRAAK within a submillisecond timescale.^[^ ^74^ ^]^ In a Xenopus oocyte membrane patch experiment, TRAAK channel opening was rapidly observed when they applied ultrasound stimulation (5 MHz). The activation mechanism was highly consistent with that induced by conventional negative‐pressure mechanical stretching. In the field of neuromodulation, ultrasound has been demonstrated to induce Ca^2^⁺ influx and neuronal firing through activation of mechanosensitive channels such as Piezo1 or members of the transient receptor potential (TRP) family. Zhu et al. systematically identified Piezo1 as the key mediator of ultrasound‐induced cellular responses, as Piezo1 knockout markedly attenuated the Ca^2^⁺ signaling response.^[^ ^75^ ^]^ Furthermore, Liao et al. discovered that Piezo1 was highly sensitive to pulse length (PL) and shear stress, with the optimal PL ranging between 10 and 100 ms, maximizing channel activation efficiency while minimizing cellular damage.^[^ ^76^ ^]^

Overall, ultrasound exhibits unique operability and promising clinical potential in membrane protein regulation, with significant progress already achieved in mechanosensitive ion channels. However, direct regulation of non‐mechanosensitive receptors, enzyme‐linked receptors, or membrane‐localized signaling proteins remains in its infancy. Future studies should focus on systematic optimization of ultrasound parameters and elucidation of protein response mechanisms to achieve broader‐spectrum and more precise functional modulation of membrane proteins.

In summary, physical stimuli such as light, temperature, magnetic fields, and ultrasound offer multilevel strategies for the programmable regulation of membrane proteins (**Table** **1**), each with distinct mechanisms and application‐specific advantages. Light‐based regulation, characterized by high spatiotemporal resolution and precise wavelength selectivity, enables real‐time control of the activity and distribution of membrane proteins at the molecular level. However, its limited tissue penetration restricts its applicability in deep physiological environments. Temperature‐based regulation generally modulates membrane protein activity via localized thermal effects or conformational shifts in thermosensitive molecular switches, offering reversibility. Nonetheless, heat diffusion may lead to nonspecific effects, requiring precise spatial control. Magnetic regulation utilizes the mechanical effect of superparamagnetic or magneto‐responsive nanomaterials to remotely and noninvasively induce aggregation of membrane protein complexes. However, potential biocompatibility and cytotoxicity issues of magnetic nanoparticles remain unresolved. In contrast, ultrasound regulates membrane proteins through multiple mechanisms, offering excellent spatiotemporal precision and controllability, though excessively high acoustic intensities may cause membrane damage or trigger cellular stress responses. Overall, magnetic field and ultrasound‐based regulation hold great promise for achieving in vivo membrane protein modulation owing to their superior tissue penetration and remote controllability.

**Table 1:** Physical technology‐mediated membrane protein regulation strategy.

Physical technology	Sensing unit	Combination unit	Membrane protein type	Cell behaviour	In vivo study	References
Uv light	Azo	Aryl diazene unit	GABA receptor	Regulation of microtubule dynamic polymerization	No	[26]
Uv light	Azo	SA	CA	Modulation of CA activity	No	[27]
Uv light	Azo	SA	CA, AChE	Universally applicable enzyme activity modulation	No	[28]
Uv light	AzoF	D7A	LRD‐7V	Reversible receptor‐protein interactions	No	[29]
Uv light	Nitrobenzene	Gu^+^ side chain	c‐Met	Regulation of the interaction between HGF and its receptor c‐Met	No	[22]
Uv light	His_6_	His_6_	Neuropeptide Y_2_ receptor	Increased dwell time of adjacent G proteins	No	[30]
NIR light	PA	Sgc8	PTK7	NIR‐triggered internalization, degradation	Yes	[31]
NIR light	PtNPs	/	GA enzyme	Rapid and reversible enzyme activity modulation	No	[37]
NIR light	GNR	PEG‐NH_2_ membrane adsorption	TREK‐1 ion channel	Photothermal activation blocking action potentials	No	[38]
NIR light	CuS NP	Anti‐TRPV1 antibody	TRPV1	Ca^2^⁺ influx, macrophage modulation	Yes	[39]
NIR light	AuNRs	Ligand	F‐actin	Recruitment of paxillin and focal adhesion proteins into focal adhesions, controlling cell migration	No	[40]
Temperature	PNIPAAm	N49C mutant streptavidin	Biotin	Temperature‐controlled binding / unbinding	No	[49]
Temperature	PNIPAAm	HER2‐targeted peptide	HER2 receptor	Inhibiting cancer cell proliferation	No	[50]
Magnetism	Magnetic beads	MHC‐Ig dimer + anti‐CD28	TCR/CD28	T cell activation, proliferation	Yes	[55]
Magnetism	Superparamagnetic nanobeads	DNP‐HSA	FcεRI receptor	Increase in intracellular Ca2^+^ concentration	No	[60]
Magnetism	Superparamagnetic nanobeads	Anti‐EGFR antibody	EGFR	EGFR activation	No	[61]
Magnetism	FMNP	Nanobody/ligand	Various (e.g., transferrin receptor)	Membrane rearrangement	No	[62]
Magnetism	Fe_3_O_4_ core + AuNP	RGD ligand	Integrin	Adhesion modulation	No	[63]
Magnetism	mEGFP	αGFP nanobody	TIAM1	Cell migration	No	[66]
Magnetism	MNP	Anti‐α5 antibody/aptamer	Integrin α5	Osteogenic signaling	No	[67]
Magnetism	Fe_3_O_4_ aggregates	RGD on AuNP	Integrin	macrophage pro‐healing	Yes	[68]
Magnetism	Zn‐doped iron oxide	Corresponding ligand	VE‐cadherin, Notch receptor	Cell fate determination, tissue development, cell adhesion, and angiogenesis	No	[69]
Magnetism	Zn‐doped iron oxide	DR4 antibody	DR4 receptor	Promotion of apoptotic signaling pathways	Yes	[70]
Ultrasound	Mechanosensitive TRAAK	/	TRAAK (K⁺ channel)	Rapid activation, mechanical gating	No	[74]
Ultrasound	Piezo1 channel	/	Piezo1	Ultrasound‐induced Ca^2^⁺ influx, mechano‐transduction	No	[75]
Ultrasound	Acoustic radiation force	/	General mechanosensitive proteins	FA formation, cytoskeletal reinforcement	Yes	[76]

## 3 Biomolecule‐Mediated Membrane Protein Regulation Strategies

### 3.1 DNA

In addition to the encoding function, DNA is also used as a key chemical material for nanodevices by virtue of its precise base pairing, programmable structure, and versatile modification capabilities.^[^ ^77^ ^]^ The currently developed DNA structures exhibit controllable sizes and shapes at the nanoscale, making them powerful tools for regulating cell membrane proteins.^[^ ^78^, ^79^, ^80^, ^81^, ^82^, ^83^, ^84^, ^85^ ^]^

#### 3.1.1 Multiple Oligonucleotide Structures

Multiple oligonucleotide structures composed of multiple oligonucleotide chains, such as oligonucleotide clusters or multi‐aptamer assemblies, represent composite systems with multivalent recognition properties. These structures can specifically bind multiple cell‐surface receptors, enabling more sophisticated regulatory functions than single‐stranded architectures. At present, a variety of strategies, from inducing receptor dimerization to precisely controlling receptor interactions, have been developed using multi‐oligonucleotide structures.

**Figure 5:**
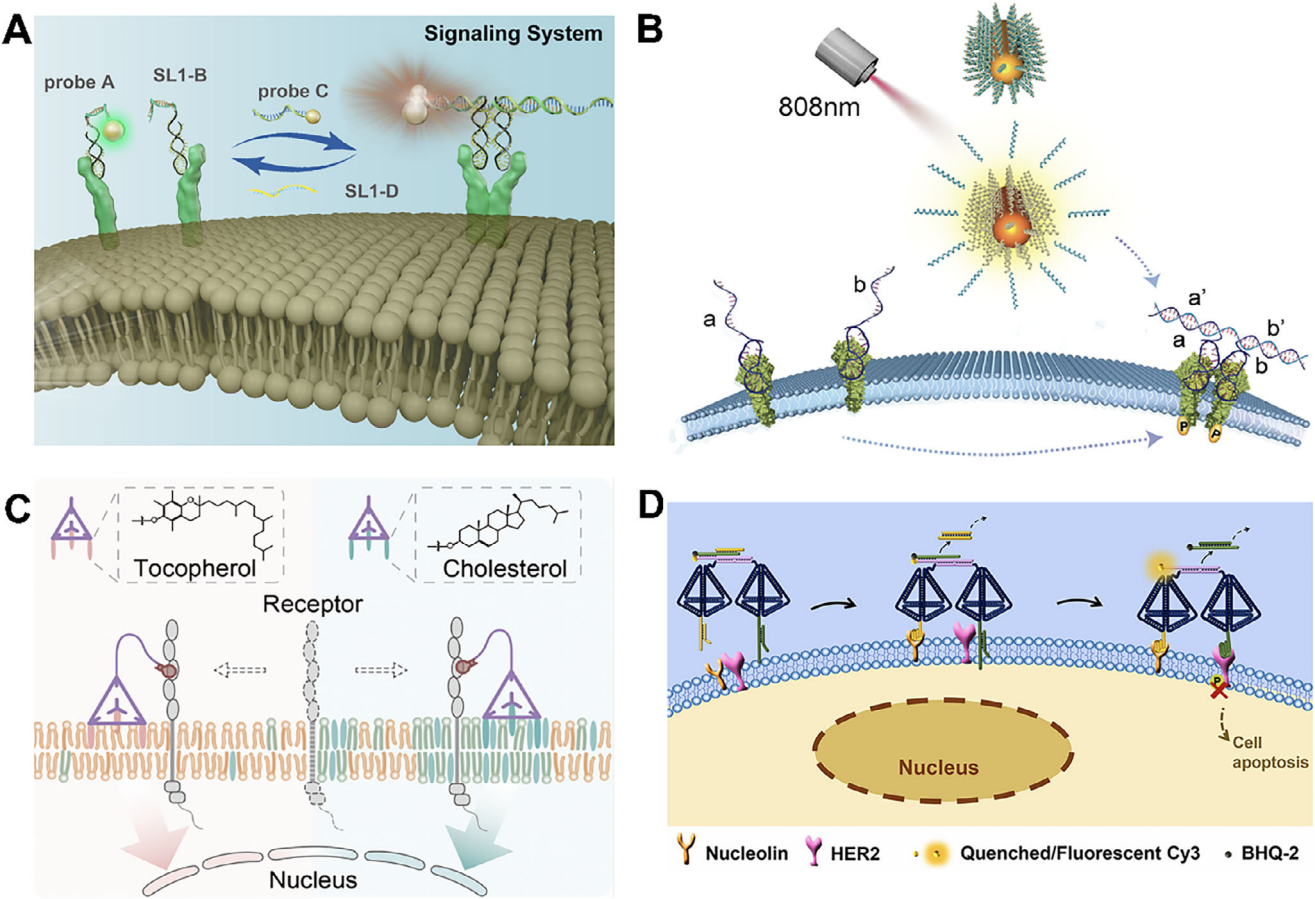
Multiple oligonucleotide structures‐ and DNA tetrahedra‐mediated membrane protein regulation strategies. A) Plasma nanoprobes programmed with DNA achieve Met receptor dimerization through hybridization‐mediated assembly. Reproduced with permission.^[^ ^87^ ^]^ Copyright 2023, Journal of the American Chemical Society. B) NIR‐triggered‐DNA agonist nanodevices on gold nanorods (NIR DA) induced receptor tyrosine kinases dimerization. Reproduced with permission.^[^ ^88^ ^]^ Copyright 2019, Nano Letters. C) Amphiphilic DNA tetrahedra functionalized with cholesterol or tocopherol vertices regulated the translocation of PTK7 protein between the liquid‐ordered and liquid‐disordered domains. Reproduced with permission.^[^ ^90^ ^]^ Copyright 2024, Journal of the American Chemical Society. D) DNA tetrahedral dimer was used for dual recognition of nucleolin and HER2 mediated spatial separation of the two membrane proteins, and inhibited HER2 activation.^[^ ^91^ ^]^ Copyright 2024, Chemical Communications.

  For example, Liang et al. designed a bispecific aptamer probe that could simultaneously recognize and bind both the Met receptor and the transferrin receptor (TfR).^[^ ^86^ ^]^ By using the aptamer as a “mechanical gripper” to grasp the two receptor proteins and the DNA linker as a “telescopic arm” to precisely regulate the receptor distance, the system enabled precise receptor regulation. More advanced protein regulation requires dynamic and reversible intervention. To this end, Xu et al. developed a plasmonic nanoprobe consisting of three components: AuNPs functionalized with single‐stranded DNA SL1‐A (probe A), which recognized Met and provided a signal output; SL1‐B, an auxiliary strand facilitating DNA hybridization; and AuNPs modified with SL1‐C (probe C), which responded to hybridization and induced Met dimerization (**Figure** **5**A).^[^ ^87^ ^]^ Probe A and SL1‐B bind to two monomeric Met receptors. Subsequent introduction of probe C initiated DNA hybridization, leading to receptor dimerization. Furthermore, adding SL1‐D, a complementary strand to SL1‐C, could trigger a strand displacement reaction, leading to the dissociation of DNA hybridization structures and restoring Met dimers into monomers, thereby achieving reversible regulation of the Met receptor. In addition, this process could be monitored in real time by tracking scattering signals from probes A and C using dark‐field microscopy. Similarly, the Wang team developed a light‐triggered DNA‐based method to induce dimerization of RTKs utilizing the photothermal effect of surface plasmon resonance (Figure 5B).^[^ ^88^ ^]^ DNA agonist nanodevices were obtained by coupling pre‐inactivated DNA agonists onto gold nanorods (AuNRs). Under NIR irradiation, the photothermal effect of AuNRs triggered the release of DNA agonists, which then hybridized with pre‐modified DNA strands on the cell membrane and assembled into DNA complexes to induce RTK dimerization, thereby activating downstream signal transduction and enhancing cell migration and proliferation. Notably, the strategies described above primarily rely on noncovalent interactions. To achieve more durable and stable control of membrane proteins, Li and colleagues reported an aptamer‐directed dual‐site photoaffinity labeling strategy.^[^ ^89^ ^]^ In this system, two aptamer probes were designed to specifically target distinct domains of membrane proteins (such as Met and PTK7), each probe was modified with an optimized photoreactive group, o‐nitrobenzyl alcohol (NB‐1). Upon UV irradiation, NB‐1 formed stable covalent bonds with adjacent lysine residues, enabling dual‐site fixation of the target proteins. Furthermore, the introduction of a complementary DNA strand induced directional aggregation of membrane proteins, effectively suppressing the phosphorylation of the Met receptor mediated by HGF and its natural activation process, and significantly reducing tumor cell migration.

#### 3.1.2 DNA Tetrahedra

DNA tetrahedra are highly stable 3D nanostructures formed by the self‐assembly of four single‐stranded oligonucleotides. Owing to their exceptional structural rigidity and precisely programmable spatial geometry, they provide an ideal scaffold for constructing multifunctional platforms to regulate membrane proteins. Compared with linear or flexible nucleic acid architectures, DNA tetrahedra exhibit superior stability and multivalent modification capability on the membrane surface. By introducing aptamers or responsive elements at the vertices or edges, DNA tetrahedra enable targeted recognition, spatial localization, and dynamic regulation of membrane proteins.

For example, the Tan team developed a multifunctional DNA nanotechnology platform based on amphiphilic DNA tetrahedra to regulate membrane protein distribution (Figure 5C).^[^ ^90^ ^]^ The DNA tetrahedron contained three hydrophobic vertices functionalized with cholesterol (Tcho3, targeting the liquid‐ordered [Lo] domain) or tocopherol (Ttcp3, targeting the liquid‐disordered [Ld] domain), while the remaining vertex was modified with the sgc8c aptamer. This design enabled dynamic translocation of the PTK7 protein between the two membrane domains through pivot‐mediated chain displacement. This DNA nanodevice could also be extended to regulate T‐cell activation by manipulating the translocation of CD45 between the Lo and Ld domains. Researchers further exploited the spatial configuration of DNA tetrahedra to achieve multitarget recognition and signal interference in membrane proteins. Zhang et al. constructed a DNA tetrahedral dimer (tetrahedral‐N and tetrahedral‐H) for separating of nucleolin and HER2 on the membranes of malignant cells (Figure 5D).^[^ ^91^ ^]^ The DNA tetrahedron was conjugated to a DNA complex composed of three strands through co‐hybridization. Among them, the Cy3‐labeled strand was positioned close to the BHQ‐2‐labeled strand, resulting in fluorescence quenching and the formation of the DNA tetrahedral dimer. Through the closed aptamers closed nucleolin aptamer and the closed HER2 aptamer extending from its two corners, nucleolin and HER2 were recognized, respectively. The interaction between BN and nucleolin led to the dissociation of the closed chains, after which the closed strand subsequently displaced the DNA chains in the connecting complex. The BHQ‐2‐labeled strand was dissociated from the Cy3‐labeled strand, resulting in fluorescence recovery. Importantly, due to the steric hindrance of the DNA tetrahedra between nucleolin and HER2, HER2 activation was inhibited, and Bax levels were upregulated, thereby inducing cell apoptosis. With advances in structural complexity, DNA tetrahedra have been increasingly employed for the dynamic and reversible regulation of membrane protein behavior. Zhao and colleagues developed a 3D allosteric DNA tetrahedron nanostructure (Apt‐HtFNA) in which the aptamer was encapsulated inside the structure in an “in” state, rendering it inaccessible to membrane proteins.^[^ ^92^ ^]^ Upon the introduction of an external DNA fuel strand (FS), the nanostructure underwent a conformational transition that exposed the aptamer in an “out” state, allowing it to bind to the target protein c‐Met and mediate its internalization into the lysosomal degradation pathway. Reversible regulation was achieved by subsequently adding an antifuel strand (AFS) to re‐hide the aptamer. This strategy could also be extended to HER2 protein degradation, demonstrating its universality and programmability.

#### 3.1.3 DNA Origami

DNA origami is a highly programmable self‐assembly technique that uses numerous short staple strands to fold a long single‐stranded DNA scaffold into predesigned nanostructures. This approach enables the fabrication of complex 3D nanostructures with precise spatial resolution and molecular arrangement. In membrane protein regulation, DNA origami structures can serve as customizable molecular scaffolds that can be modified with aptamers, signaling modules, or responsive motifs, achieving precise control over membrane proteins.

**Figure 6:**
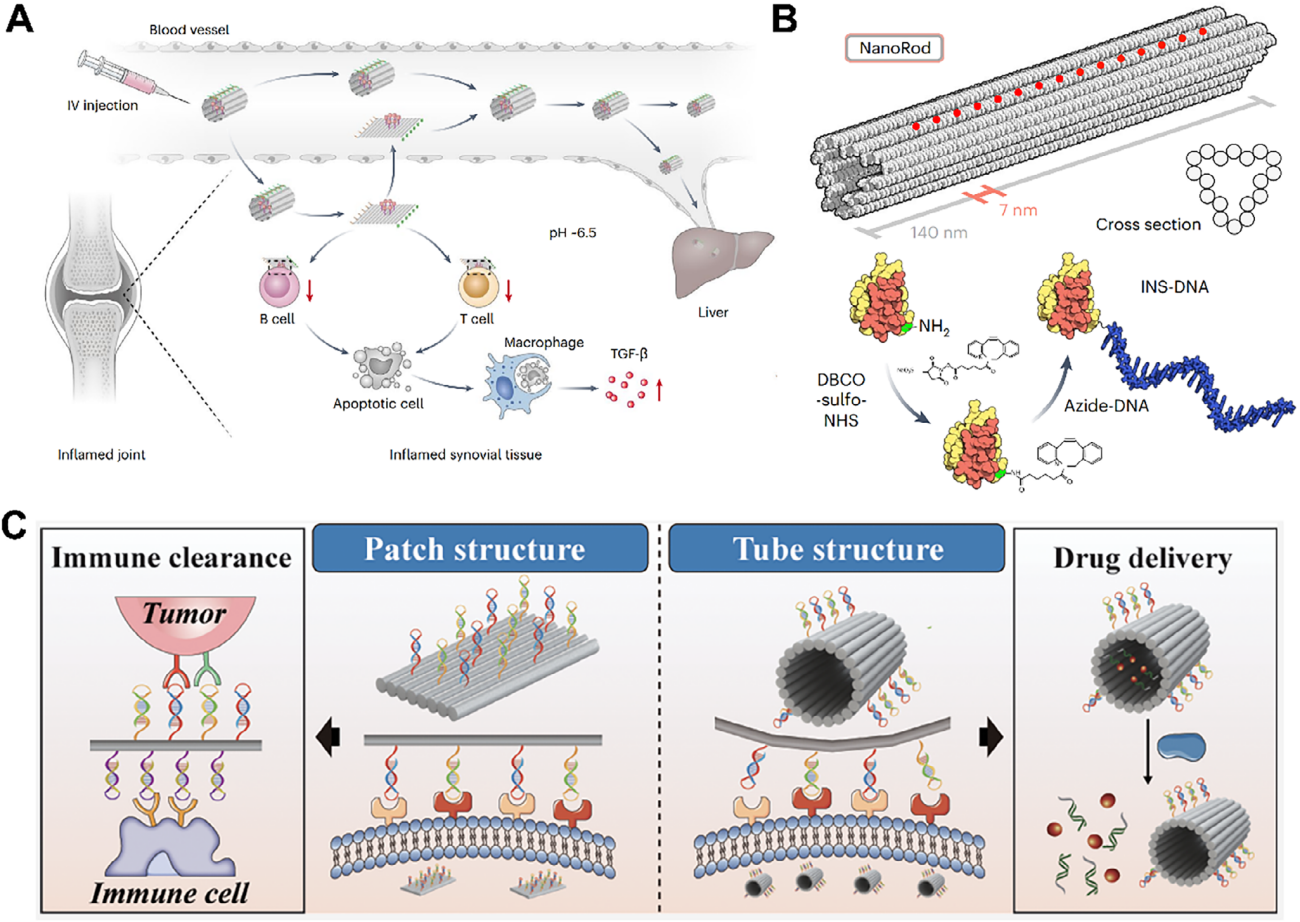
DNA origami‐mediated membrane protein regulation strategies. A) A pH‐responsive 2D DNA origami incorporating an i‐motif switch induced CD95 receptor clustering. Reproduced with permission.^[^ ^94^ ^]^ Copyright 2024, Nature Materials. B) A multivalent insulin‐ligand nanoarray constructed on a rod‐shaped DNA origami scaffold enhanced multivalent activation of the insulin receptor. Reproduced with permission.^[^ ^96^ ^]^ Copyright 2024, Nature Nanotechnology. C) Planar and tubular DNA origami nanostructures modified with multivalent aptamers for immune recognition of tumor cells and targeted drug delivery. Reproduced with permission.^[^ ^99^ ^]^ Copyright 2024, Journal of the American Chemical Society.

For instance, Zeng et al. constructed a DNA origami‐based nanoplatform termed DoriVac, capable of precisely controlling CpG spacing to regulate Toll‐like receptor 9 (TLR9) receptor clustering and signal polarization.^[^ ^93^ ^]^ CpG oligonucleotides were arranged on a square DNA origami with tunable intervals ranging from 2.5 to 7.0 nm, among which a 3.5 nm spacing facilitated optimal TLR9 dimerization and signal transduction, significantly enhancing IL‐12 secretion and antigen presentation. Compared with free CpG or conventional carriers, DoriVac achieved stronger CD8⁺ T cell activation and antitumor immunity at markedly lower dosages. Furthermore, Ling Li and colleagues engineered a pH‐responsive 2D DNA origami nanoplatform for regulating the spatial clustering of the CD95 receptor (**Figure** **6**A).^[^ ^94^ ^]^ The hexagonal ligand array was geometrically matched to the CD95 receptor cluster. By incorporating i‐motif conformational switches, the platform enabled pH‐dependent ligand exposure under acidic conditions. This mechanism triggered high‐order receptor aggregation and activated caspase‐8, ultimately achieving localized immunosuppression and inflammation control. This design exemplifies microenvironment‐selective regulation of receptor activation through DNA origami‐based nanostructures. With the advancement of DNA origami design and operability, researchers have begun integrating physical and chemical stimuli into these systems. Li et al. developed a DNA origami‐gold nanorod hybrid nanoheater that enables localized membrane heating under NIR irradiation.^[^ ^95^ ^]^ The platform employed aptamers targeting either lipid raft or non‐raft regions to anchor the nanostructure on the specific membrane domains. NIR‐triggered local temperature elevation altered lipid packing and membrane tension, thereby modulating integrin clustering and cell migration dynamics. In a murine wound‐healing model, the system facilitated migration and tissue regeneration, demonstrating the synergistic potential between DNA origami structures and external physical modulation.

In addition, DNA origami has been employed to quantitatively regulate receptor signaling strength via multivalent ligand design. For example, Spratt et al. developed a rod‐shaped origami nanostructure composed of 18 helices, on which multiple DNA‐conjugated insulin ligands were spatially organized on one face (Figure 6B).^[^ ^96^ ^]^ Controlled increases in ligand valency markedly enhanced insulin receptor retention on the membrane and signaling intensity, thereby improving cellular metabolic responses. This work highlighted the capability of DNA origami to quantitatively regulate multivalent signaling processes. In addition, Wang et al. employed a DNA‐origami scaffold to position TRAIL‐mimicking peptides with a spatial precision of 2‐10 nm, thereby constructing a hexagonal multivalent ligand nanoarray capable of binding to the death receptor DR5 on the cell membrane and inducing apoptosis.^[^ ^97^ ^]^ When the spacing between the TRAIL‐mimicking peptides was ≈5 nm, they efficiently induced DR5 clustering on the membrane surface, promoting the recruitment and activation of Caspase‐8 and subsequently triggering downstream apoptotic signaling pathways. In contrast, when the spacing increased to 11 nm or more, receptor clustering and Caspase‐8 activation were significantly reduced, leading to a marked decrease in apoptosis efficiency. Similarly, Smyrlaki et al. designed a soluble multivalent Jagged1 (ligands of the Notch receptor) nanopattern by precisely arranging Jag1‐Fc fusion ligands, comprising the extracellular domain of Jag1 fused to an Fc fragment, on a DNA origami scaffold anchored to the cell membrane via cholesterol modifications.^[^ ^98^ ^]^ The platform effectively induced activation of Notch receptors and triggered downstream signaling without requiring external mechanical force. Furthermore, Hu et al. fabricated planar (DNPs) and tubular (DNTs) DNA origami nanostructures, which were further modified with multivalent aptamers (mvAp‐DNPs and mvAp‐DNTs) (Figure 6C).^[^ ^99^ ^]^ The planar constructs primarily retained on the membrane surface to enhance receptor recognition, whereas the tubular counterparts more effectively induced receptor‐mediated endocytosis, serving as immune‐bridging platforms to promote macrophage‐mediated tumor recognition and elimination.

#### 3.1.4 Hybridization Chain Reaction (HCR)‐Based DNA Structures

HCR is an enzyme‐free nucleic acid amplification process that enables the programmable assembly of DNA polymers through the hybridization of hairpin structures under an external triggering signal. This mechanism endows the system with excellent dynamic responsiveness and signal amplification capability, providing a promising strategy for constructing controllable, multilevel DNA nanonetworks on cell membranes. HCR‐based architectures can integrate functional modules, such as aptamers, responsive sequences, or signal elements, to achieve spatial clustering and activity regulation of membrane proteins, and can also self‐assemble in situ to form stable DNA scaffolds for studying membrane domain reconstruction and signal transduction.

**Figure 7:**
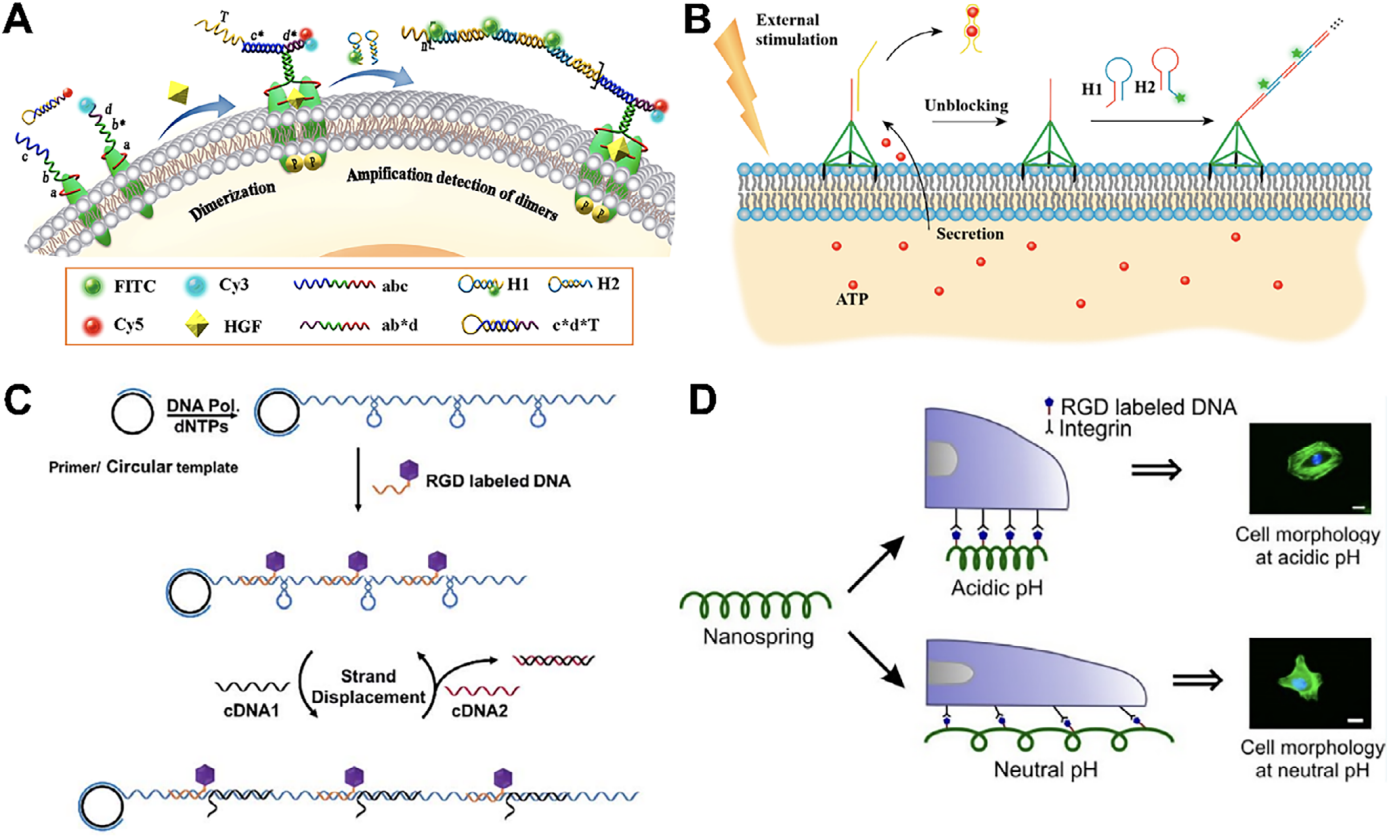
Hybridization chain reaction (HCR)‐ and rolling circle amplification (RCA)‐based DNA structures‐mediated membrane protein regulation strategies. A) A dynamic DNA nanodevice based on aptamer recognition and proximity‐induced HCR enabled amplified detection and real‐time imaging during c‐Met receptor dimerization. Reproduced with permission.^[^ ^100^ ^]^ Copyright 2021, Journal of the American Chemical Society. B) A DNA tetrahedron modified with three cholesterol anchors and ATP‐responsive vertex anchored to the cell membrane via cholesterol, and ATP triggered the H1/H2 HCR, thereby enabling precise regulation of specific cells. Reproduced with permission.^[^ ^103^ ^]^ Copyright 2018, Analytical Chemistry. C) RCA‐constructed DNA nanosprings bearing RGD‐modified multivalent ligands undergo cDNA‐mediated hairpin opening to contract the nanospring, thereby promoting integrin clustering and adhesion formation. Reproduced with permission.^[^ ^104^ ^]^ Copyright 2017, Chemical Science. D) pH‐driven DNA origami nanosprings employing i‐motif folding/unfolding to reversibly modulate regulated the distance between integrin receptors. Reproduced with permission.^[^ ^105^ ^]^ Copyright 2021, Bioconjugate Chemistry.

For instance, Wang et al. designed a dynamic DNA nanodevice based on aptamer recognition and proximity‐induced HCR, which specifically recognized membrane receptors c‐Met (**Figure** **7**A).^[^ ^100^ ^]^ The proximity effect generated by the binding of neighboring probes activated a “contact‐dependent trigger” probe, releasing an HCR initiator that drives the cascade polymerization of signal probes H1 and H2. This system enabled amplified reaction and real‐time imaging during receptor dimerization. Furthermore, Wang and coworkers promoted unidirectional aggregation of PTK7 receptors, inducing DNA self‐assembly on the surface of living cells.^[^ ^101^ ^]^ The DNA HCR system consisted of five components: trigger chain, hairpin 1 (HP1), hairpin 2 (HP2), inhibitor, and activator (completely complementary to the inhibitor), in which the inhibitor and trigger complexed to prevent their reaction with HP1 until activation. Bridging the DNA strand with the sgc8c aptamer could anchor it to the PTK7 receptor on the cell surface. Subsequently, the activator was added to replace the inhibitor and initiate the chain reaction. DNA self‐assembly led to the PTK7 receptors aggregation, thus reducing the cell migratory. In addition, heterogeneous clustering of two different membrane proteins could be achieved by changing the DNA aptamers attached to the two hairpin chains. In another study, Su et al. employed metabolic signals as external triggers to regulate membrane organization.^[^ ^102^ ^]^ They constructed an ATP‐triggered, HCR‐driven DNA nanodevice, which was anchored to the cell membrane via a Cholera toxin B subunit‐specific aptamer (CT916). Upon ATP binding, the hairpin structure of chain I was sequentially opened, driving the hybridization of H1 and H2 on the membrane to form a high‐molecular‐weight DNA network. This network subsequently rearranged lipid raft components through electrostatic interactions, enriched cholesterol and membrane proteins, and induced membrane phase separation. Ultimately, these changes led to the redistribution of the transmembrane receptor CD44 across membrane domains, thereby inhibiting cell migration and epithelial‐mesenchymal transition. Similarly, Tan's group constructed a dynamic membrane protein simulation platform based on a DNA tetrahedron, in which three vertices were anchored to the cell membrane through cholesterol moieties, and an ATP aptamer probe was introduced at another vertex (Figure 7B).^[^ ^103^ ^]^ In the presence of ATP, the exposure of the aptamer probe triggered a cascading hybridization reaction between single‐stranded DNA monomers H1 and H2, generating a traceable DNA polymer for signal transmission. Furthermore, by modifying H2 with a formyl group and expressing the corresponding receptor on K562 cells, intercellular connections between PC12 and K562 cells were achieved. When the photosensitizer chlorin e6 was incorporated into H1, localized apoptosis could be induced under visible light irradiation, thereby enabling precise regulation of specific cells.

#### 3.1.5 Rolling Circle Amplification (RCA)‐Based DNA Structures

RCA is a highly efficient isothermal nucleic acid amplification technique that produces ultralong single‐stranded DNA with numerous repeated sequences. RCA products can be precisely programmed to arrange aptamer or ligand units along the DNA scaffold, enabling controllable regulation of the spatial distribution and clustering of cell‐surface receptors, thereby influencing signal transduction and cellular behavior.

For example, the Zhang team developed DNA nanosprings for reversibly regulating the distance between integrin receptors (Figure 7C).^[^ ^104^ ^]^ Long DNA scaffolds were prepared by RCA, and hairpin structures were encoded in circular templates to mimic spring‐like configurations. RGD‐labeled DNA was then hybridized with the long DNA scaffold to generate multivalent ligands. With the addition of the complementary DNA1 (cDNA1), the hairpin structure on the scaffold opened and hybridize with cDNA1, forming a relatively rigid double‐stranded DNA sequence. The DNA nanospring adopted a contracted state, promoting the aggregation of integrin receptors and the formation of regular adhesive spots on the substrate. The addition of cDNA2 to replace cDNA1 resulted in the reformation of hairpin structures, an increased distance between integrin receptors, and cell morphology change from normal to having many cell protrusions. The researchers further incorporated environment‐responsive elements into the RCA‐constructed nanospring to achieve intelligent control over receptor distribution. In a related approach, Mao et al. designed a pH‐responsive DNA origami nanospring that regulated cell movement by promoting the aggregation of integrin receptors (Figure 7D).^[^ ^105^ ^]^ Nanosprings were modified with RGD domains to target integrin receptors, and adjacent piers of the nanosprings were aggregated together using i‐motif motifs. At slightly acidic pH, the i‐motif folding promoted the coiling of DNA nanosprings and inhibited the movement of HeLa cells. At neutral pH, the unfolding of i‐motif allowed cells to recover mechanical motion as the nanospring unfolded. Similarly, the Zhang team developed a pH‐responsive interlocking DNA nanospring (iDNS) to regulate the nanoscale distribution of CD3 and CD28 receptors.^[^ ^106^ ^]^ Compared with conventional DNA structures, the interlocking circular DNA nanostructure of iDNS provided a more stable scaffold for ligand assembly. IDNS consisted of three types of circular DNA (C1, C2, and C3) and CD3 and CD28 receptor ligands. The circular DNA template chains could be assembled together through nucleic acid base complementary hybridization sequences. Both C2 and C3 encode two i‐motif sequences, and cDNA of the i‐motif sequences hybridized with iDNS to put it in an extended state. In melanoma tumors with low pH (pH 6.5), disruption of the i‐motif sequence induced iDNS contraction, thereby promoting T cell proliferation and enhancing antitumor activity.

#### 3.1.6 Other DNA Nanostructures

In addition to the aforementioned DNA nanostructures, Tan and colleagues designed a tweezer‐like DNA nanosystem capable of controllable conformational switching through strand displacement reactions, thereby precisely modulating the lateral spacing between CD28 receptors during T‐cell activation.^[^ ^107^ ^]^ In the initial state, single‐stranded DNA self‐hybridized to form a hairpin structure that brought the two arms closer together. Upon introduction cDNA, single‐stranded DNA extended to form a rigid double‐stranded structure and opened the tweezers; adding DNA that was completely complementary to cDNA could restore the hairpin structure. This structure employed aptamers that specifically bound to CD28 as recognition units, and achieved reversible switching of aptamers opening and closing through chain substitution, thereby dynamically regulating the distance between CD28 receptors to promote or weaken T‐cell activation.

**Figure 8:**
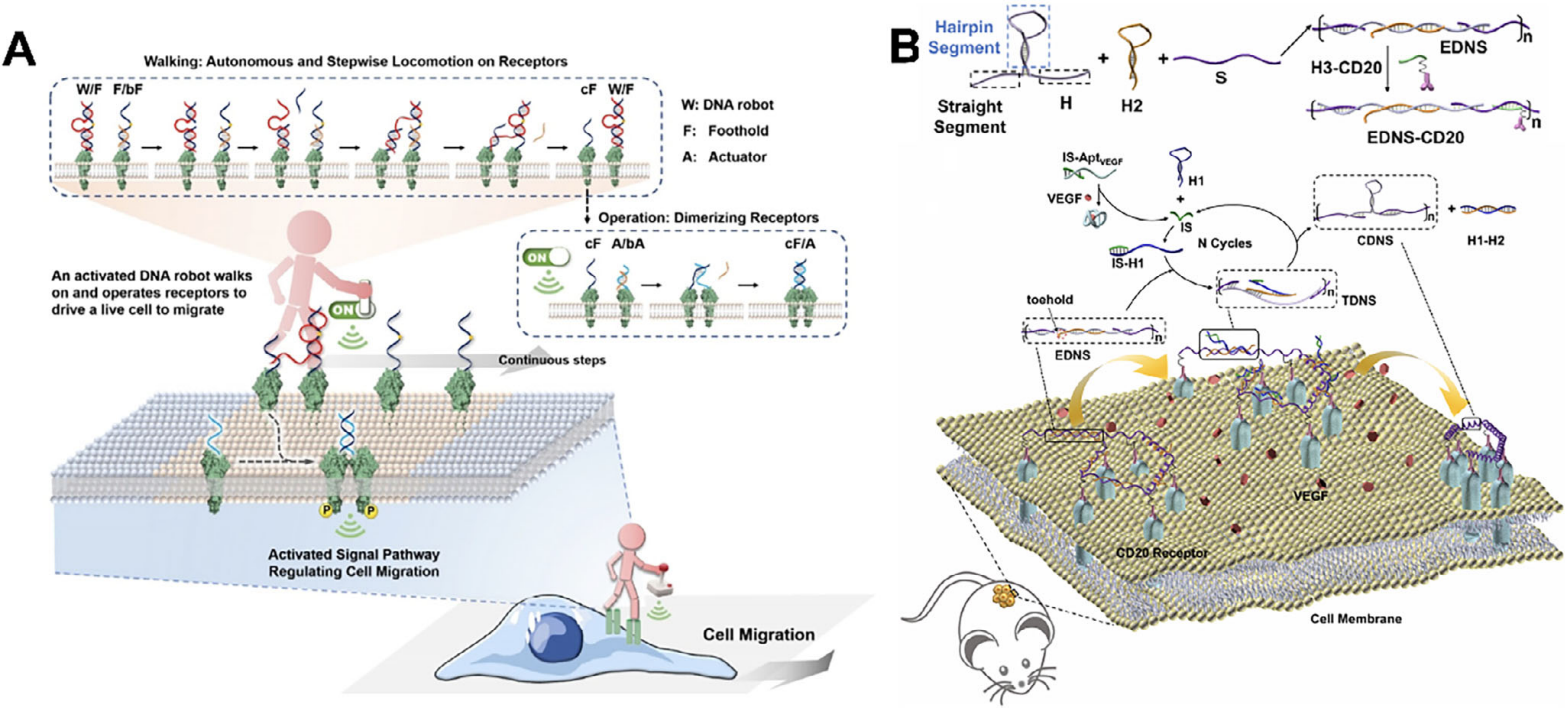
Other DNA nanostructures‐mediated membrane protein regulation strategies. A) DNA nanorobot sequentially displaced blocking strands on receptor‐anchored footholds and actuators, thereby driving Met receptor dimerization and enhancing cell migration. Reproduced with permission.^[^ ^109^ ^]^ Copyright 2021, Angewandte Chemie International Edition. B) VEGF‐triggered conformational contraction of an extended DNA nanostructure, where CD20 antibodies were anchored via thiol‐modified H3 DNA strands, induced CD20 receptor clustering. Reproduced with permission.^[^ ^110^ ^]^ Copyright 2023, Journal of the American Chemical Society.

Researchers have further developed DNA nanorobotic systems capable of precisely adjusting receptor spacing and even “walking” on the cell membrane to realize dynamic and spatially controlled manipulation of membrane proteins. These DNA nanorobots are programmable molecular devices composed of recognition, response, and actuation modules. They can perform functions such as cargo release, conformational rearrangement, or receptor clustering in response to specific stimuli (e.g., ligand binding, pH change, or enzymatic activity), providing novel strategies for spatial regulation of membrane proteins. For example, the Liu team developed a DNA nanorobotic for the dedimerization of Met receptors. This DNA nanorobotic consisted of a hairpin chain (H), adapter Met 1 (A1), and adapter Met 2 (A2).^[^ ^108^ ^]^ The two ends of the H chain hybridized with A1 and A2, respectively, while the H chain self‐hybridized to form a stem‐loop structure. The proximity between A1 and A2 increased, and the migratory ability of cells with Met receptor dedimerization was enhanced. After adding an open chain (Op), the stem‐loop structure hybridized with Op to form a rigid double‐stranded complex (NR/O), thereby increasing the distance between A1 and A2 and promoting Met receptor dimerization. In the presence of a closed chain (Cl), it could also transition back to a tighter configuration. In addition, Nie et al. proposed a DNA nanorobotic design that could walk on fluid cell membranes and induce Met receptor dimerization to enhance cell migration (**Figure** **8**A).^[^ ^109^ ^]^ It consisted of a single‐legged walking robot (W), multiple receptor‐anchored feet (F), and multiple receptor‐anchored actuators (A). W contained a DNA enzyme catalytic core, and flanking recognition arms that hybridized with complementary regions on F. F and anchored Met receptors via specific aptamer motifs and were pre‐blocked by complementary short chains bF and bA. During operation, W replaced bF on F to form W/F double strands and generate cF, which then bound A to displace bA, producing acF/A double strands anchored to Met receptor dimerization and induced self‐phosphorylation. This DNA nanorobotic autonomously moved across multiple footholds, repeatedly generating cF/A dimers to accumulate receptor dimers and amplify signaling. To move beyond externally driven mechanisms, Liu's group incorporated endogenous cellular signaling triggers to construct a self‐feedback DNA regulatory system (Figure 8B).^[^ ^110^ ^]^ In this design, CD20 antibodies were conjugated to thiol‐modified DNA strands (H3‐SH) to form H3‐CD20, which hybridized with complementary fragments on an extended DNA nanostructure (EDNS) to yield the EDNS‐CD20 complex. The VEGF‐binding aptamer recognized Raji cell‐secreted VEGF, releasing an “initiator” strand that hybridized with H1 to trigger conformational changes in the DNA nanostructure. Subsequent strand displacement with H2 restored the hairpin structure, transforming the EDNS into a contracted conformation that induced CD20 receptor aggregation, thereby activating apoptosis and achieving selective killing of Raji cells.

In summary, various DNA nanostructures exhibit significant potential for membrane protein regulation (**Table** 2). Static rigid structures, such as DNA tetrahedra and DNA origami, offer highly predictable geometries and nanoscale spatial precision, enabling precise modulation of receptor aggregation and signal transduction. However, their rigidity and relatively large size limit flexibility in dynamic membrane environments, potentially reducing membrane binding efficiency. Incorporating flexible linkers into these static structures can enhance membrane adaptability while maintaining spatial precision. In contrast, dynamic and responsive structures, including oligonucleotide‐based assemblies, HCR constructs, RCA systems, and DNA nanorobots, possess flexible assembly and triggering mechanisms, allowing dynamic regulation of membrane receptors, real‐time signal amplification, and visualized operation. Nevertheless, their complex reaction kinetics, potential chain entanglement, amplification errors, and operational challenges may compromise regulatory precision and hinder in vivo applications. Overall, the development of DNA nanotechnology is trending toward integrating the precise spatial control of static structures with the responsiveness of dynamic architectures, forming controllable and precise platforms for membrane protein regulation. For in vivo applications, further optimization of DNA structural stability, membrane adaptability, and immunological safety will be essential.

**Table 2:** Biomolecule‐mediated membrane protein regulation strategies.

Types of biomolecules	Combination unit	Membrane protein types	Cellular behavior	In vivo study	References
Multiple oligonucleotide structures	Apt‐Met, Apt‐TfR	Met receptor and TfR	Inhibit cell migration	No	[86]
Multiple oligonucleotide structures	SL1‐A, SL1‐B	Met receptor	Dynamic monitoring of transmembrane protein dimerization	No	[87]
Multiple oligonucleotide structures	RTKs aptamer	RTKs	Enhance cell migration	Yes	[88]
Multiple oligonucleotide structures	NB‐1 dual aptamers	Met/PTK7	Receptor aggregation, suppress activation	No	[89]
DNA tetrahedra	Cholesterol/tocopherol + sgc8c	PTK7, CD45	Domain translocation	No	[90]
DNA tetrahedra	Apt‐HER2/Apt‐Nucleolin	HER2, Nucleolin	Apoptosis	No	[91]
DNA tetrahedra	Apt‐cMet/Apt‐HER2	c‐Met/HER2	Controlled internalization	No	[92]
DNA origami	CpG spacing 2.5‐7 nm	TLR9	DC activation	Yes	[93]
DNA origami	i‐motif ligand array	CD95	Caspase‐8 activation	No	[94]
DNA origami	Apt‐AuNR	Integrin	Migration	Yes	[95]
DNA origami	Multivalent insulin ligands	Insulin receptor	Enhanced signaling	No	[96]
DNA origami	TRAIL‐mimic peptide	DR5	Apoptosis	No	[97]
DNA origami	Jagged1‐Fc	Notch receptor	Activation	No	[98]
DNA origami	Multivalent aptamer	Multiple receptors	Macrophage activation	Yes	[99]
DNA origami	H1/H2 + Met aptamer	Met	Dimerization imaging	No	[100]
HCR	Sgc8c	PTK7 protein	Driving the unidirectional aggregation of homogeneous and heterogeneous membrane proteins on the cell surface	No	[101]
HCR	CTB‐aptamer	CD44	EMT suppression	No	[102]
HCR	ATP‐triggered	Membrane proteins	Intercellular link	No	[103]
RCA	RGD‐labeled DNA	Integrin receptor	Cell morphological change	No	[104]
RCA	RGD‐labeled DNA	Integrin receptor	Inhibit the movement of HeLa cells	No	[105]
RCA	CD3/CD28 ligands	CD3/CD28	T cell proliferation	Yes	[106]
Other DNA nanostructures	CD28 aptamer	CD28	Regulate T cell activation	No	[107]
Other DNA nanostructures	A1, A2	Met receptor	Promotion of cell migration	No	[108]
Other DNA nanostructures	Met aptamer	Met receptor	Promotion of cell migration	No	[109]
Other DNA nanostructures	Anti‐CD20 antibody	CD20 receptor	Induction of Raji cell apoptosis	Yes	[110]
Peptides	G7‐RGD	CD3 receptor	T cell activation	No	[121]
Peptides	PAD‐1	EGFR	Cancer cell death	Yes	[122]
Peptides	BP‐FFVLK‐HER2loop	HER2	Inhibit signaling, apoptosis	Yes	[123]
Peptides	CD81‐binding peptide	CD81	Inhibit migration	Yes	[124]
Peptides	Pep‐20	CD47 and CD24 receptor	Enhanced macrophage phagocytosis	Yes	[125]
Peptides	CCK2R peptide + ALP module	CCK2R	PDT, pyroptosis	Yes	[126]
Peptides	DR5L	DR5	Promote cell apoptosis	No	[127]
Protein	TMV‐SPPEPS	Integrin	Clustering/signaling	No	[128]
Protein	AaLS‐TRAIL‐EGFR	EGFR/DR	Apoptosis	Yes	[129]
Protein	RIPR‐PD1	CD45 and PD‐1 receptor	Enhanced T cell activity	Yes	[130]
Protein	NK and CD45 antibody	NK and CD45 receptor	Enhanced NK and T cell function	Yes	[131]
Protein	DR5 antibody	DR5 receptor	Induction of cell apoptosis	No	[132]
Protein	mAb‐CMA peptide	EGFR, HER2, PD‐L1	Lysosomal degradation	Yes	[133]

### 3.2 Peptides and Proteins

Beyond DNA, peptides and proteins provide a powerful and versatile toolkit for modulating membrane protein function. Their intrinsic biocompatibility, degradability, together with hydrophobic segments, lipidation, or transmembrane‐mimicking motifs, enable more natural anchoring within the membrane environment, making them highly adaptable platforms for precise membrane protein regulation.

#### 3.2.1 Peptides

As synthetic biological molecules, peptides can be obtained either through genetically encoded sequences or chemically synthesized libraries, thereby offering opportunities to edit and customize modular structures. Their excellent good biocompatibility, ability to bypass biological barriers, deep tissue penetration, and environmental responsiveness endow them with substantial potential in membrane protein regulation.^[^ ^111^, ^112^, ^113^, ^114^, ^115^, ^116^, ^117^, ^118^, ^119^, ^120^ ^]^

A prominent strategy has been to design peptides that self‐assemble upon binding target membrane proteins, thereby inducing receptor clustering and subsequent regulation. The assembly‐enhancement mechanism is based on anchoring self‐assembling peptides to specific proteins, stabilizing the peptide conformation, and lowering the activation energy for assembly. For example, Wang et al. developed a programmable peptide conjugate, in which the anti‐CD3 peptide specifically bound to the CD3 receptor on T cells, and the RGD peptide targeted the integrin αVβ3 receptor on tumor cells. The G7 segment (with the amino acid sequence GNNQQNY) served as a self‐assembly module.^[^ ^121^ ^]^ After anti‐CD3‐G7‐RGD binding to the CD3 receptor on T cells, the peptide conformation was stabilized, and the activation energy for self‐assembly decreased, thereby promoting the oligomerization of the anti‐CD3 peptide. In co‐culture experiments, T cells treated with anti‐CD3‐G7‐RGD were observed to cluster significantly around tumor cells. This result suggested that CD3 oligomerization and subsequent T cell activation could effectively induce T cell‐mediated lysis of cancer cells. Similarly, the Wang team designed a cyclic peptide PAD‐1 composed of a self‐assembling peptide backbone, a flexible connector that adjusted the hydrophilicity of the system, and an EGFR recognition moiety.^[^ ^122^ ^]^ PAD‐1 could recognize the membrane protein EGFR and prevent EGFR signaling, and induce cancer cell death mediated by assembly‐induced aggregation through multivalent interactions. PAD‐1 oligomers could recognize and bind to EGFR, inhibit EGFR signal transduction, and subsequently initiate cellular uptake through endocytosis. The accumulation of PAD‐1 in lysosomes led to self‐assembly, which triggered EGFR nanofiber aggregation and induced lysosomal membrane permeabilization, ultimately inducing tumor cell apoptosis. In another example, Zhang et al. developed an intelligent supramolecular peptide (BP‐FFVLK‐YCDGFYACYMDV) comprising three functional modules: i) a biphenyl (BP) segment with aggregation‐induced emission (AIE) properties that acted as a hydrophobic core to induce nanofibril formation; ii) FFVLK, the reverse sequence of the KLVFF β‐sheet‐forming domain from amyloid‐β (Aβ) peptide; and iii) YCDGFYACYMDV, a disulfide‐cyclized HER2‐binding loop.^[^ ^123^ ^]^ Upon binding HER2 on cancer cells, the peptide transformed in situ into nanofibrils, disrupted HER2 dimerization, blocked downstream signaling, suppressed nuclear transcription of proliferation and survival genes, and ultimately triggered apoptosis. Zhao's group used genetic codon degeneracy to rationally design site‐specific affinity peptides that regulated intercellular clustering of CD81.^[^ ^124^ ^]^ The peptides bound the extracellular loop (ECL) of CD81 with high affinity, stabilizing the peptide‐protein complex and inducing conformational rearrangement. This conformational change propagated across the transmembrane helices, relocating CD81 from a dispersed membrane distribution to the cell‐cell contact interface, where it formed localized clusters. This spatial reorganization enhanced intermolecular interactions and transmembrane signaling of CD81, ultimately suppressing tumor cell migration. In vivo mouse studies further demonstrated that the peptide effectively inhibited lung metastasis of highly invasive triple‐negative breast cancer.

**Figure 9:**
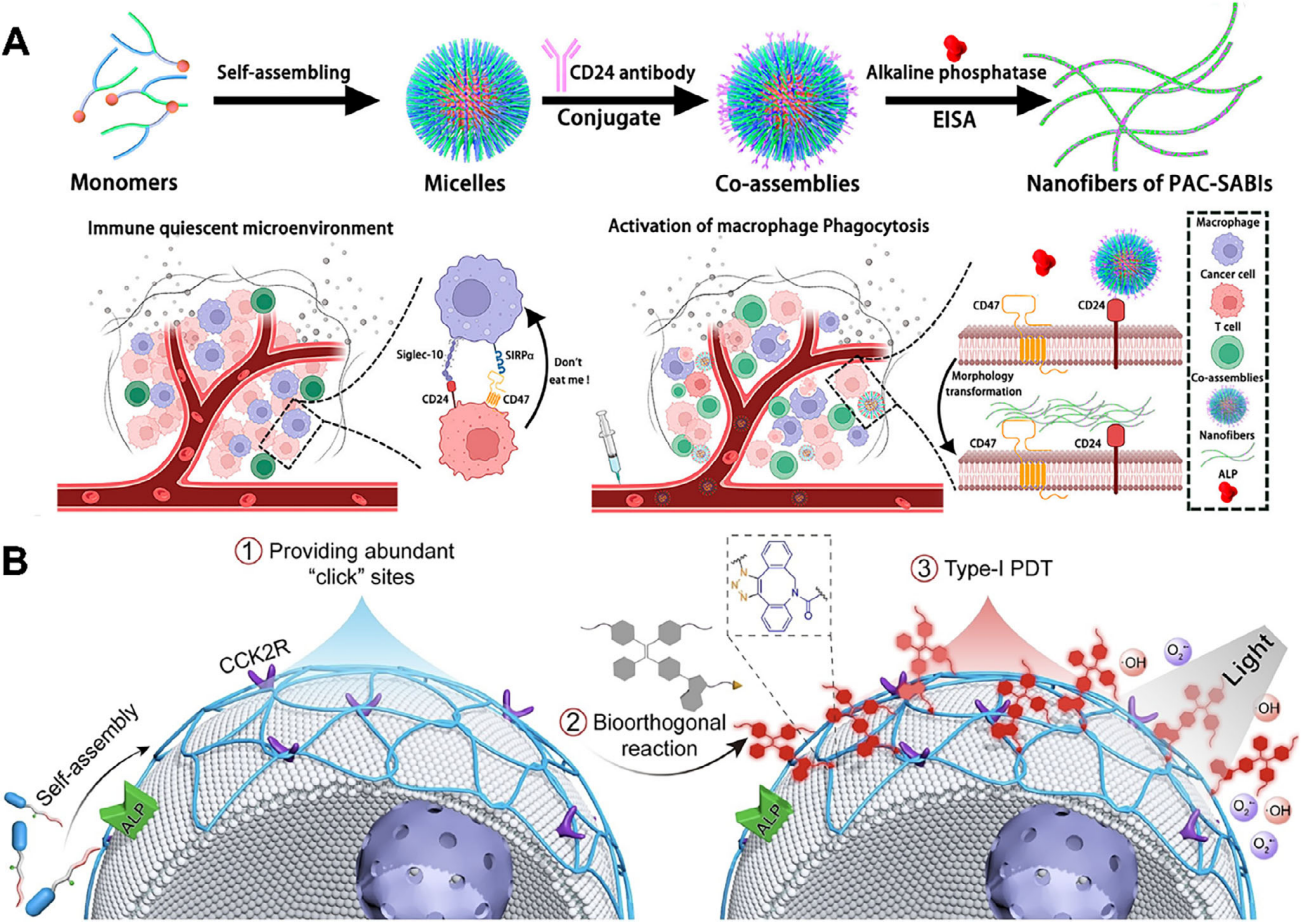
Peptide molecules‐mediated membrane protein regulation strategies. A) Dual‐targeting peptide (PAC‐SABI) for CD47 and CD24 underwent alkaline phosphatase‐triggered conformational rearrangement that drove its self‐assembly into a membrane‐wrapping nanofiber network to block both receptor pathways. Reproduced with permission.^[^ ^125^ ^]^ Copyright 2024, Nature Communications. B) A membrane‐anchored peptide responsive to both alkaline phosphatase (ALP) and the cholecystokinin‐2 receptor (CCK2R) self‐assembled into nanofibrils on cancer cells and enriched aggregation‐induced emission photosensitizers for type‐I photodynamic therapy (PDT) to induce cell death. Reproduced with permission.^[^ ^126^ ^]^ Copyright 2025, Angewandte Chemie International Edition.

Another strategy relies on enzyme‐catalyzed conformational transitions. The Cai team designed a dual‐target inhibitory molecule (PAC‐SABI) containing tyrosine residues in a peptide scaffold (Lys Leu Val Phe Phe), which achieved higher specificity and efficacy by incorporating the natural curved topology of the cell membrane into the structural design (**Figure** **9**A).^[^ ^125^ ^]^ The peptide segment (Pep‐20) that could bind to CD47 can be assembled into micelles in aqueous solution, and then connected to anti‐CD24 antibodies to actively target cancer cells overexpressing CD24. Then, CD24 antibodies, binding together with alkaline phosphatase catalysis, induced peptide rearrangement to form a nanofiber network that wrapped around the cancer cell membrane, thereby exerting a synergistic macrophage immune regulatory effect. The experiment showed that PAC‐SABI could enhance the phagocytosis of macrophages in vitro and in vivo by blocking CD47 and CD24 signal transduction, and promote the anti‐tumor response of breast cancer and pancreatic cancer mouse models. This approach highlights the potential of enzyme‐instructed supramolecular self‐assembly on the membrane surface to create a physical barrier that blocks receptor function.

In addition, enzyme‐ and receptor‐dual triggered membrane‐anchored self‐assembling peptide platforms have been used for membrane functionalization and photosensitizer enrichment. Ji et al. reported a dual‐targeting peptide for alkaline phosphatase (ALP) and cholecystokinin 2 receptor (CCK2R), which self‐assembled into nanofibrils on cancer cell membranes (Figure 9B).^[^ ^126^ ^]^ These structures then bioorthogonally enriched aggregation‐induced emission (AIE) photosensitizers on the membrane surface for type‐I photodynamic therapy (PDT), inducing pyroptosis and immunogenic cell death (ICD).

Beyond single‐component systems, multicomponent peptide co‐assembly offers more refined approaches to constructing peptide‐protein complexes. Jiang and coworkers used multiple synthetic peptides to directly and noncovalently link receptors on the cell surface to regulate cell behavior.^[^ ^126^ ^]^ Three types of cell interface co‐assemblies with different ligand distributions were designed by regulating receptor spacing. This was achieved through controlled peptide crosslinking and specific peptide dimerization, employing different numbers of spacer peptides to generate modular spacers. Simultaneously, the cyclic DR5 targeting ligand was conjugated to the C‐terminus of the peptide, allowing it to anchor to cancer cell surface receptors and regulate cancer cell apoptosis.

#### 3.2.2 Protein

Compared with peptides, proteins are suitable for constructing multivalent ligands, artificial receptors, or bispecific molecules capable of precisely modulating membrane protein clustering and signal transduction, provide strong structural foundations and functional potential for programmable membrane protein control.

**Figure 10:**
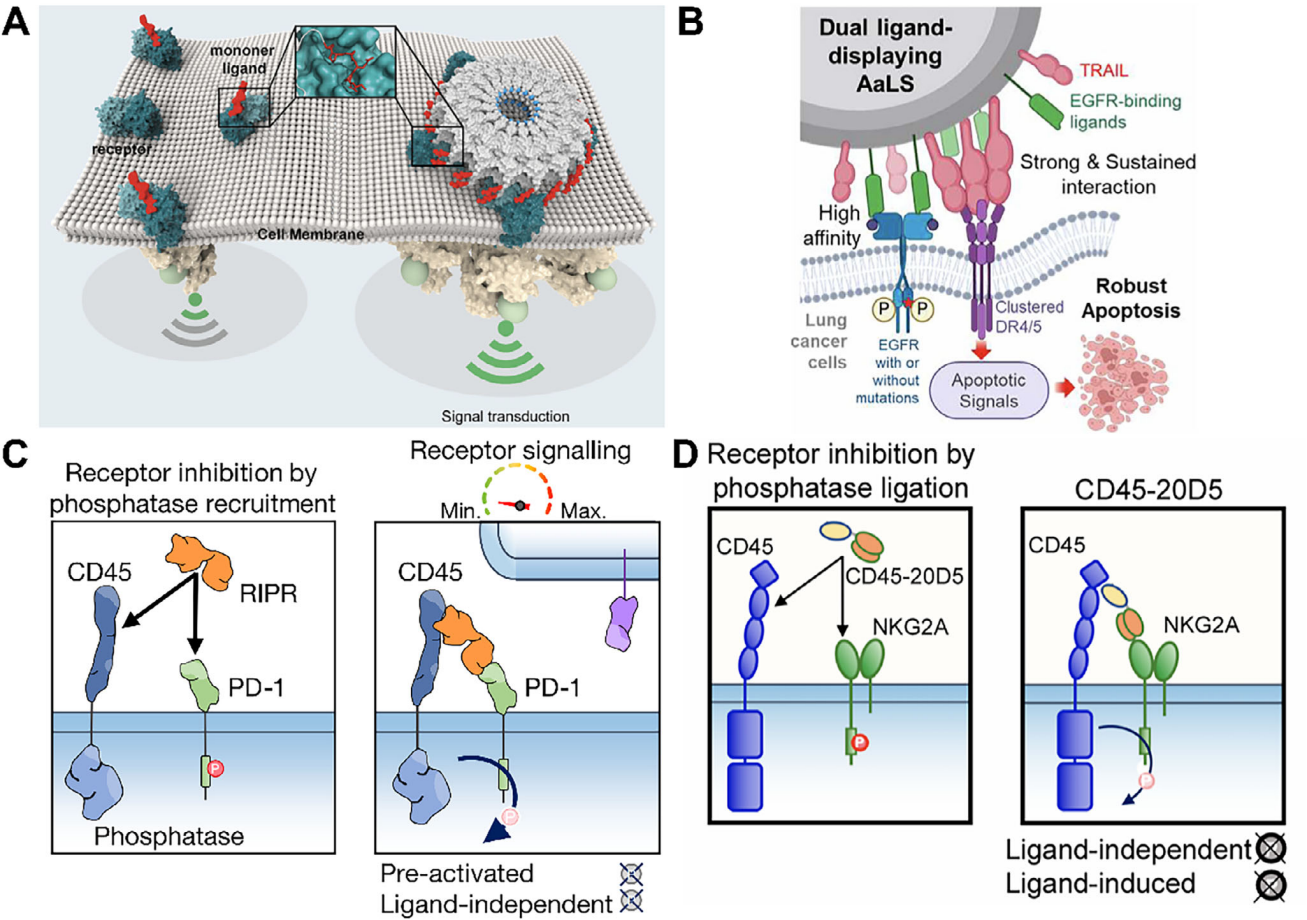
Protein molecules‐mediated membrane protein regulation strategies. A). A nanodisc constructed from tobacco mosaic virus (TMV) coat proteins was functionalized with multivalent integrin‐binding peptides to induce integrin clustering. Reproduced with permission.^[^ ^127^ ^]^ Copyright 2025, Angewandte Chemie International Edition. B) Protein nanoparticles based on Aquifex aeolicus lumazine synthase (AaLS) were engineered to display both tumor necrosis factor‐related apoptosis‐inducing ligand (TRAIL) and epidermal growth factor receptor (EGFR) ligands, enabling selective targeting of EGFR‐overexpressing cancer cells and enhancing TRAIL‐mediated apoptotic signaling. Reproduced with permission.^[^ ^128^ ^]^ Copyright 2025, ACS Applied Materials & Interfaces. C) The bispecific antibody RIPR‐PD1 recruited CD45 and PD‐1 through a double antigen binding arm to inhibit PD‐1’s intracellular tetrameric signaling. Reproduced with permission.^[^ ^129^ ^]^ Copyright 2021, Nature. D) Bispecific antibodies induced the proximity between inhibitory NKR and CD45 to enhance NK cell killing. Reproduced with permission.^[^ ^130^ ^]^ Copyright 2022, ACS Synthetic Biology.

Direct use of natural proteins or enzymes as structural templates enables the construction of highly ordered and programmable nanoplatforms for membrane protein regulation. Wang Qiangbin's group engineered a nanodisc based on tobacco mosaic virus (TMV) coat protein, whose 34 regularly arranged surface sites allowed precise display of peptides or ligands (**Figure** **10**A).^[^ ^127^ ^]^ A multivalent integrin‐binding construct, TMV‐SPPEPS, bound approximately seven integrin receptors simultaneously, drawing dispersed integrins onto the nanodisc to induce local clustering and activate downstream FAK/MAPK signaling. Kim et al. used aquifex aeolicus lumazine synthase (AaLS) to engineer target‐specific protein nanoparticles simultaneously displaying multiple TRAIL molecules and EGFR‐binding ligands (Figure 10B).^[^ ^128^ ^]^ This design enhanced selective targeting of EGFR‐overexpressing lung adenocarcinoma and squamous carcinoma cells, promoted TRAIL clustering on the cell surface, improved TRAIL‐death receptor (DR) binding, strengthened particle‐cell adhesion, and amplified TRAIL‐mediated apoptotic signaling.

Bispecific antibodies provide another strategy for precise membrane receptor control by simultaneously recognizing two targets and reconfiguring their spatial relationships. Garcia and coworkers developed a bispecific antibody RIPR‐PD1, to suppress PD‐1 immune receptor signaling by forcibly recruiting CD45 phosphatases (Figure 10C).^[^ ^129^ ^]^ The RIPR‐PD1 was constructed by fusing anti‐CD45 antibody with anti‐PD‐1 antibody. CD45 was recruited to the vicinity of PD‐1 through a dual antigen‐binding arm, achieving direct dephosphorylation of PD‐1 tyrosine residues within the cell, thereby inhibiting PD‐1 signaling and enhancing T‐cell activity. Similarly, the proximity between inhibitory natural killer (NK) receptors (NKRs) and CD45 could exploit the dephosphorylation activity of CD45 to inhibit NKR‐mediated inhibitory signaling (Figure 10D).^[^ ^130^ ^]^ Ren et al. used bispecific anti‐CD45‐NKR molecules to enhance NK cells and T cells’ function.^[^ ^131^ ^]^ Although multivalent antibodies could increase binding affinity and signal transduction, there was still a lack of generalizable methods for forming precisely oriented antibody assemblies with controlled valence states.

Computationally designed proteins provide a means to precisely and modularly regulate membrane protein function by assembling antibodies into structures with defined geometries and valencies. The Baker team designed an antibody cage (AbC) by rigidly connecting the antibody constant region binding module with a cyclic oligomer via a helical spacer domain, which arranged the symmetry axes of the dimer antibody and the cyclic oligomer at different angles, forming a dihedral or polyhedral structure (such as tetrahedra, octahedra, icosahedra).^[^ ^132^ ^]^ The AbCs promoted large‐scale DR5 receptor aggregation and enhanced signal transduction by forming 2D arrays on the cell surface.

Antibody‐peptide conjugates offer another route for membrane protein degradation. For example, Shao et al. developed an antibody‐peptide conjugation strategy based on chaperone‐mediated autophagy (CMA), termed Ab‐CMA, which enabled efficient degradation of membrane proteins such as EGFR, programmed death‐ligand 1 (PD‐L1), and HER2.^[^ ^133^ ^]^ This system consisted of two components: a targeting module and a degradation module. The targeting module was a monoclonal antibody that specifically recognized the extracellular domain of the membrane protein, while the degradation module was a CMA signal peptide (KFERQKILDQRFFE) that could be recognized by heat shock cognate 70 kDa protein (Hsc70), guiding the target protein into the lysosome. After Ab‐CMA bound to the membrane protein, the signal peptide was recognized by Hsc70 and subsequently transported into the lysosome through the lysosome‐associated membrane protein type 2A (LAMP2A)‐mediated CMA pathway, thereby achieving targeted protein degradation.

A nanodisc constructed from tobacco mosaic virus (TMV) coat proteins was functionalized with multivalent integrin‐binding peptides to induce integrin clustering. Reproduced with permission.^[^ ^127^ ^]^ Copyright 2025, Angew Chem Int Ed Engl. B) Protein nanoparticles based on Aquifex aeolicus lumazine synthase (AaLS) were engineered to display both tumor necrosis factor‐related apoptosis‐inducing ligand (TRAIL) and epidermal growth factor receptor (EGFR) ligands, enabling selective targeting of EGFR‐overexpressing cancer cells and enhancing TRAIL‐mediated apoptotic signaling. Reproduced with permission.^[^ ^128^ ^]^ Copyright 2025, ACS Appl Mater Interfaces. C) The bispecific antibody RIPR‐PD1 recruited CD45 and PD‐1 through a double antigen binding arm to inhibit PD‐1′s intracellular tetrameric signaling. Reproduced with permission.^[^ ^129^ ^]^ Copyright 2021, Nature. D) Bispecific antibodies induced the proximity between inhibitory NKR and CD45 to enhance NK cell killing. Reproduced with permission.^[^ ^130^ ^]^ Copyright 2022, ACS Synthetic Biology.

Overall, peptides and proteins possess inherent biocompatibility and therefore offer clear advantages over DNA nanostructures for in vivo applications. Peptides exhibit high conformational flexibility and can undergo enzyme‐mediated or receptor‐triggered self‐assembly to drive receptor clustering, However, their stability and target selectivity remain relatively limited. Proteins, by contrast, feature well‐established targeting capabilities and a combinatorial space of structural domains that enable multivalent binding, bispecific molecular architectures, and precisely engineered geometric control through computational design. In addition, compared with DNA and peptides, proteins generally display lower immunogenicity and greater in vivo stability, making them a clinically translatable class of platform for regulating membrane protein function.

## 4 Summary and Prospect

In summary, membrane proteins play a central role in maintaining cellular homeostasis and orchestrating physiological processes, particularly receptor recognition, transmembrane signal transduction, and the regulation of cellular behaviors. In recent years, membrane protein modulation has emerged as a promising avenue for therapeutic intervention. Here, we summarized membrane protein regulation strategies, including light/temperature/magnetism/ultrasound‐based physical methods and DNA/peptides/proteins‐based biomolecular strategies.

Despite substantial progress, several challenges remain. For example, photo‐responsive physical regulation approach is constrained by limited tissue penetration, hindering its effective in vivo application. By contrast, magnetic field‐ and ultrasound‐based methods have already demonstrated efficacy in vivo animal models. Although physical‐responsive materials exhibit excellent controllability and stimulus specificity, their biosafety profiles with local inflammation or organ toxicity remain a major challenge. In contrast, biomolecular materials offer superior biocompatibility and intrinsic molecular programmability. DNA nanostructures offer a well‐defined sequence‐encoded framework that enables nanoscale geometric control, whereas peptides and proteins afford conformational flexibility and membrane affinity that closely resemble native biological systems. These properties allow them to effectively and precisely regulate membrane proteins. Nevertheless, their in vivo stability, complex operability, and high production cost presently limit operational robustness and large‐scale application. Furthermore, the current understanding of the functional mechanisms of membrane proteins remains relatively limited. The biological processes involving membrane proteins are typically complex and variable. A comprehensive elucidation of their roles in various physiological and pathological states is crucial for developing more precise and effective therapeutic strategies.

By optimizing the design of nucleic‐acid and protein materials, along with their targeting elements and delivery strategies, future research can enhance their in vivo stability and treatment specificity, facilitating their clinical translation. In addition, one feasible direction for future research is the development of multimodal synergistic regulation platforms, which integrate external physical stimuli (such as light, magnetic fields, ultrasound) or microenvironment‐responsive triggers (such as enzymes and pH) with DNA‐, peptide‐, or protein‐based programmable modules to achieve precise spatiotemporal control of membrane proteins. Meanwhile, such platforms can also be employed to investigate the effects of different stimuli on membrane protein signaling, providing novel tools for understanding complex cellular behaviors.

In conclusion, although the current strategy for regulating membrane proteins has inherent risks and challenges, the modular design enabled by synthetic biology, the material stability and versatility endowed by nanotechnology, and the high spatiotemporal monitoring capabilities of advanced bioimaging techniques are collectively driving the field toward greater precision, intelligence, and translational potential. In addition, a deeper understanding of membrane protein structure and functional mechanisms, combined with the integration of interdisciplinary technologies, is expected to facilitate the development of safe and efficient membrane protein regulation strategies, further expanding their practical applications in disease research and therapy.

## Conflict of Interest

The authors declare no conflict of interest.
